# The WAVE3/β-catenin oncogenic signaling regulates chemoresistance in triple negative breast cancer

**DOI:** 10.1186/s13058-023-01634-3

**Published:** 2023-03-22

**Authors:** Wei Wang, Priyanka S. Rana, Vesna Markovic, Khalid Sossey-Alaoui

**Affiliations:** 1 Department of Medicine, MetroHealth Medical Center, Cleveland, OH 44109 USA; 2 Case Western Reserve University School of Medicine, Case Western Reserve University, Cleveland, OH 44016 USA; 3 Case Comprehensive Cancer Center, Case Western Reserve University, Cleveland, OH 44106 USA

## Abstract

**Background:**

Metastatic breast cancer is responsible for the death of the majority of breast cancer patients. In fact, metastatic BC is the 2nd leading cause of cancer-related deaths in women in the USA and worldwide. Triple negative breast cancer (TNBC), which lacks expression of hormone receptors (ER-α and PR) and ErbB2/HER2, is especially lethal due to its highly metastatic behavior, propensity to recur rapidly, and for its resistance to standard of care therapies, through mechanisms that remain incompletely understood. WAVE3 has been established as a promoter of TNBC development and metastatic progression. In this study, we investigated the molecular mechanisms whereby WAVE3 promotes therapy-resistance and cancer stemness in TNBC, through the regulation of β-catenin stabilization.

**Methods:**

The Cancer Genome Atlas dataset was used to assess the expression of WAVE3 and β-catenin in breast cancer tumors. Kaplan–Meier Plotter analysis was used to correlate expression of WAVE3 and β-catenin with breast cancer patients’ survival probability. MTT assay was used to quantify cell survival. CRISPR/Cas9-mediated gene editing, 2D and 3D tumorsphere growth and invasion assays, Immunofluorescence, Western blotting, Semi-quantitative and real-time quantitative PCR analyses were applied to study the WAVE3/β-catenin oncogenic signaling in TNBC. Tumor xenograft assays were used to study the role of WAVE3 in mediating chemotherapy resistance of TNBC tumors.

**Results:**

Genetic inactivation of WAVE3 in combination of chemotherapy resulted in inhibition of 2D growth and 3D tumorsphere formation and invasion of TNBC cells in vitro, as well as tumor growth and metastasis in vivo. In addition, while re-expression of phospho-active WAVE3 in the WAVE3-deficient TNBC cells restored the oncogenic activity of WAVE3, re-expression of phospho-mutant WAVE3 did not. Further studies revealed that dual blocking of WAVE3 expression or phosphorylation in combination with chemotherapy treatment inhibited the activity and expression and stabilization of β-catenin. Most importantly, the combination of WAVE3-deficiency or WAVE3-phospho-deficiency and chemotherapy suppressed the oncogenic behavior of chemoresistant TNBC cells, both in vitro and in vivo.

**Conclusion:**

We identified a novel WAVE3/β-catenin oncogenic signaling axis that modulates chemoresistance of TNBC. This study suggests that a targeted therapeutic strategy against WAVE3 could be effective for the treatment of chemoresistant TNBC tumors.

**Supplementary Information:**

The online version contains supplementary material available at 10.1186/s13058-023-01634-3.

## Significance

Identification of a novel WAVE3/β-Catenin oncogenic signaling axis that regulates chemoresistance may provide new therapeutic strategies for the treatment of TNBC by targeting WAVE3 and/or its downstream effectors.

## Introduction

According to the latest statistics from the American Cancer Society [1], breast cancer (BC) is the number one disease affecting women in the USA, with more than 280,000 estimated new cases, while metastatic BC ranks number 2 as the cause of cancer-related deaths of women, annually accounting for more than 43,000 deaths [1]. BC is a heterogeneous disease that is comprised of at least 5 genetically different subtypes [2, 3] and more than 10 molecular subtypes [4, 5]. Among these subtypes, triple negative BC (TNBC), which lacks estrogen and progesterone hormone receptors (ER and PR) expression and Her2 amplification, accounts for 15–20% of all breast cancers. Compared to other BC subtypes, TNBC is a very aggressive cancer, in part due to its higher prevalence at a young age, its propensity to rapidly recure and metastasize, and its resistance to standard of care therapies [6]. In fact, the lack of FDA-approved therapies to specifically target TNBC tumors further aggravates the aggressiveness and the poor clinical outcome of this disease [7].

WAVE3, like all members of the WASP verprolin-homologous family of proteins, was originally identified as a regulator of actin cytoskeleton and its associated cell migration phenotype, through the regulation of membrane ruffles at the leading edge of migrating cells downstream of Rho and Rac small GTPases (reviewed in [8]). The last decade has, however, witnessed an explosion of published studies linking WAVE3 to the pathogenesis of several cancers, including BC (reviewed in [9, 10]). The oncogenic activity of WAVE3 has now been established as a major driver of the invasion–metastasis cascade in TNBC through the regulation of several hallmarks of cancer, including the epithelial-mesenchymal transition (EMT) and cancer stem cell (CSC) phenotype [11], angiogenesis [12], and apoptosis [13]. Several of these oncogenic activities of WAVE3 are believed to be mediated by the phosphorylation of WAVE3 at specific tyrosine residues [11, 14]. Given the association of the TNBC phenotype with therapy resistance, and the role of WAVE3 as a major driver of the TNBC disease, the function of WAVE3 in the regulation of chemoresistance has not been investigated, as did the role of β-catenin in this oncogenic pathway. β-Catenin has been well established as a major regulator of several complex physiological processes, such as embryonic development, maintenance of the stem cell phenotype, wound healing, and tissue homeostasis [15]. Dysregulated β-catenin signaling has also been shown to be associated with tumor progression, metastasis, and therapy resistance in several cancers, including TNBC [16]. Given the parallels between WAVE3 and β-catenin in the regulation of chemoresistance in breast cancer and other tumors, we sought to investigate the underlying molecular mechanisms that link these two major drivers of chemoresistance in TNBC. In the present study, we show that WAVE3 plays a major role mediating chemoresistance of TNBC cells in vitro and tumors, in vivo. We show that loss of WAVE3 expression or phosphorylation leads to the sensitization of TNBC cells and tumors to chemotherapy. Mechanistically, the WAVE3-mediated regulation of chemoresistance was found to be through the regulation of the CSC phenotype and through the stabilization of β-catenin.

Our data established a novel WAVE3/β-catenin signaling axis that drives chemo-resistance of TNBC, and provide the impetus for developing novel potential therapeutic options that target WAVE3 and/or β-catenin for the treatment of therapy-resistant TNBC tumors.

## Materials and methods

### Cell culture and reagents

TNBC Cell lines were acquired from the American Type Culture Collection (ATCC; Manassas, VA), and maintained according to the manufacturer’s established protocols. Cell lines were also routinely authenticated by short tandem repeat DNA fingerprinting analysis. WAVE3-deficient (W3-KO) cells were generated using two different and verified sgRNAs targeting the human *WAVE3* and the mouse *Wave3* genes by lentiviral transduction as described previously [11]. A scrambled (SCRAM) sgRNA was used as negative control [11]. Generation of the WAVE3-KO cells overexpressing wildtype (WT) or phosphomutant (Y4) were described previously [17]. GFP-tagged WAVE3 constructs were generated as described previously [17]. The GFP-recombinant vector or the empty GFP expression control vector was used for stable transfections using standard protocols. We used the following reagents: Doxorubicin, docetaxel and cisplatin, purchased from Sigma (St. Louis, MO) as powders, were dissolved in DMSO and used as described previously [12]. For proteasome inhibition, cells were treated with GM6001 (Sigma) at 20 µM for 24 h. Gel electrophoresis reagents were from Bio-Rad (Hercules, CA).

### Antibodies

Rabbit anti-WAVE3 (1:1000) and mouse anti- β-catenin (1:1000) were from Cell Signaling Technologies (Danvers, MA). Goat horseradish peroxidase-conjugated anti-mouse IgG (1:5000) and goat horseradish peroxidase-conjugated anti-rabbit IgG (1:5000) from Calbiochem (San Diego, CA); and Alexa 488-conjugated anti-rabbit IgG and Alexa 568-conjugated anti-mouse IgG are from Invitrogen (Waltham, MA). Vecta-shield with 4′,6-diamidino-2-phenylindole was from Vector Laboratories (Newark, CA).

### MTT assays

3-(4,5-dimethylthiazol-2-yl)-2,5-diphenyltetrazolium bromide (MTT) assay was performed following standard protocol. For each BC cell line, 5 × 10^3^ cells were seeded into 96-well culture plates for overnight followed by treatment with either DMSO (0.1%) or the chemotherapeutic agents as indicated for 2 days.

### Three-dimensional tumorsphere cultures

3D multi-spheroid formation and 3D spheroid invasion assays were performed as described previously [18].

### Western blotting

Western blotting assays were performed according to standard protocols. β-Actin, Lamin B or α-Tubulin were used as loading standards. ChemiDoc MP Imaging system (Bio-Rad) was used for image acquisition of developed gels. Signal intensity of WB bands was quantitatively analyzed by ImageJ software, as described previously [19] and according to the parameters described in ImageJ user guide (http://rsbweb.nih.gov/ij/docs/guige/146.html).

### In vivo studies

Parental, WAVE3-deficient (W3-KO), WAVE3-deficient overexpressing wild-type WAVE3 (W3-WT), or phospho-mutant WAVE3 (W3-Y4) MDA-MB-231 cells (10^6^ cells per mouse, *n* = 5) or 4T1 cells (10,000 cells per mouse, *n* = 5) were implanted into the mammary fat pads of 6–8-week-old female NSG or Balb/C mice, respectively. Both the left and the right mammary fat pads were injected, resulting in 10 tumors per group. For the chemotherapy treatments, once the tumors became palpable (~ 100 mm^3^), mice were randomized into control and experimental groups (*n* = 5 mice per group) and treated with either the diluent (e.g., 0.1% DMSO), cisplatin (1 mg/kg i.p) or doxorubicin (1 mg/kg i.p.), twice a week for 4 weeks. Tumor growth was monitored by twice weekly measuring tumor volume with digital Vernier calipers. At the endpoint, tumors were collected and fixed with 4% paraformaldehyde. Tumors were also weighed prior to fixation. All animal studies were performed under protocols approved by the Institutional Animal Care and Use Committee.

### Immunofluorescence

Tumors and lung tissues were harvested and snap-frozen in optimal cutting temperature (OCT) medium (Sakura Finetek; Torrance, CA), and 8-μm sections were prepared. Tumor sections were stained with the following antibodies: rabbit anti-WAVE3 and mouse anti-β-Catenin, followed by Alexa 488 anti-mouse or Alexa 568-conjugated goat anti-rabbit IgG. Lung sections were stained with hematoxylin (Vector Laboratories) and eosin (Fisher Scientific Co.; Kalamazoo, MI) following the manufacturer’s protocol. Confocal or bright-field imaging microscopy (Leica, Wetzlar, Germany) was used to capture the images and ImagePro Plus Capture and Analysis software (Media Cybernetics; Rockville, MD) was used to analyze the stained sections from 10 to 15 independent fields/sections.

### Semi-quantitative and real-time quantitative PCR

Total RNA was extracted from cancer cell lines using TRIzol reagent (Invitrogen, Camarillo, CA) following the manufacturer’s instructions. Qt-RT-PCR was performed, as described previously [20]. qPCR Primer Assays for were obtained from Qiagen. GAPDH was used as internal control. Oligonucleotide primers used for qRT-PCR were from Integrated DNA Technologies (Coralville, IA), were described in [14].

### Statistical analysis

Experiments were performed in triplicate and analyzed using the Student’s *t*-test. In calculating two-tailed significance levels for equality of means, equal variances were assumed for the two populations. Results were considered significant at *p* < 0.05.

## Results

### Loss of WAVE3 expression or its phosphorylation sensitize TNBC cell to chemotherapeutics

**Fig. 1:**
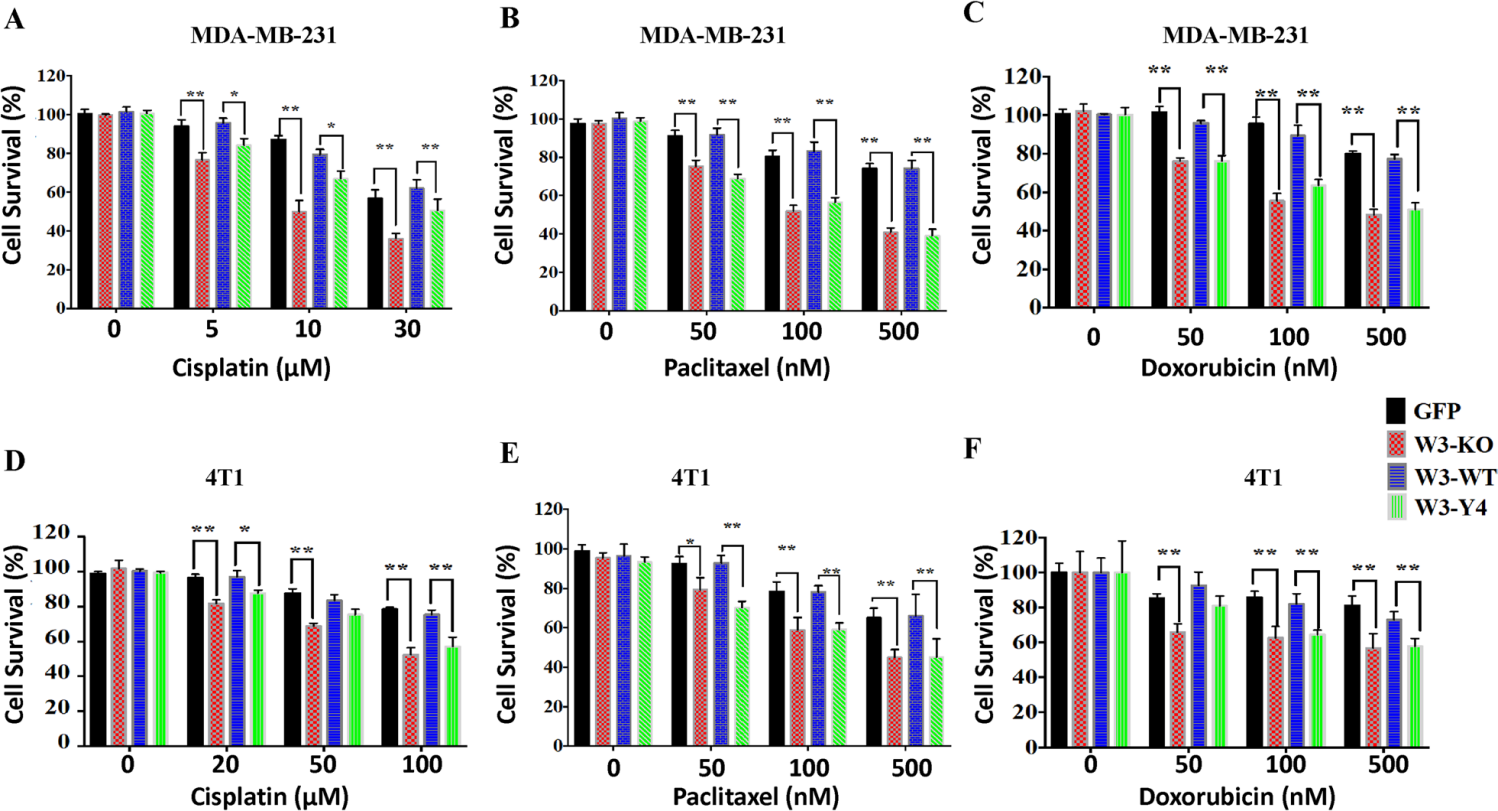
Loss of WAVE3 expression or its phosphorylation sensitize TNBC cells to chemotherapeutics. Parental MDA-MB-231 (**A**–**C**) or 4T1 (**D**–**F**) cells (GFP), their WAVE3-deficient derivatives (W3-KO), or the W3-KO expressing either wildtype (W3-WT) or phospho-mutant (W3-Y4) WAVE3 were treated with increased concentrations of cisplatin (**A** and **D**), paclitaxel (**B** and **E**) or doxorubicin (**C** and **F**) for 2 days followed by MTT assay to determine cell viability. Data are the mean ± SD (*n* = 3, *p < 0.05; **p < 0.01, Student’s t test). Data shown is representative of three independent experiments

Our previously published data reported on the role of WAVE3 and its phosphorylation at specific tyrosine residues in the progression and metastasis of BC tumors [14, 17, 18]. We have also shown that loss of WAVE3 expression sensitizes TNBC cell lines to chemotherapeutics [12]. The extent to which WAVE3 phosphorylation regulates resistance to chemotherapy has however not been investigated. Accordingly, we assessed the effect of loss of WAVE3 phosphorylation on cell survival in the presence of cisplatin, doxorubicin, and paclitaxel, three chemotherapeutic drugs that are widely used for the treatment of TNBC tumors. Generation of the phospho-mutant WAVE3 (W3-Y4) at tyrosine residues Y151, Y248, Y337 and Y486 (Additional file 1: Fig. S1A) has been described and thoroughly tested in our previous publications [17, 18], as was the generation of WAVE3-deficient BC cell lines, including MDA-MB-231 and 4T1, using CRISPR/Cas9 mediated gene knock-out [11, 14, 18]. Additional file 1: Fig. S1B shows an almost complete loss of expression of WAVE3 (W3-KO) in MDA-MB-231 cells using CRISPR/Cas9-mediated gene knockout, while Additional file 1: Fig. S1C shows re-expression of wildtype (W3-WT) or phosphomutant (W3-Y4) GFP-WAVE3 fusion proteins. Loss of WAVE3 expression in MDA-MB-231 or 4T1 TNBC cells resulted in a significant (*p* < 0.001) decrease in cell viability after treatment with cisplatin (Fig. 1A, D), paclitaxel (Fig. 1B, E) or doxorubicin (Fig. 1C, F) at concentrations as low as 5 nM with cisplatin and as low as 50 nM with doxorubicin and paclitaxel. Re-expression of wildtype (W3-WT) in the W3-KO cells restored resistance to these drugs and cell viability to levels observed with the control (GFP) cells. Re-expression of phosphomutant WAVE3 (W3-Y4), on the other hand, not only failed to restore cell viability, but resulted in inhibition of cell growth to levels comparable to those seen in the W3-KO cells. Thus, both expression of WAVE3 and its phosphorylation are required for the acquisition of TNBC cell lines to resistance to chemotherapy.

### Loss of WAVE3 expression or its phosphorylation in combination to chemotherapeutics inhibits tumorsphere formation and invasion in vitro

**Fig. 2:**
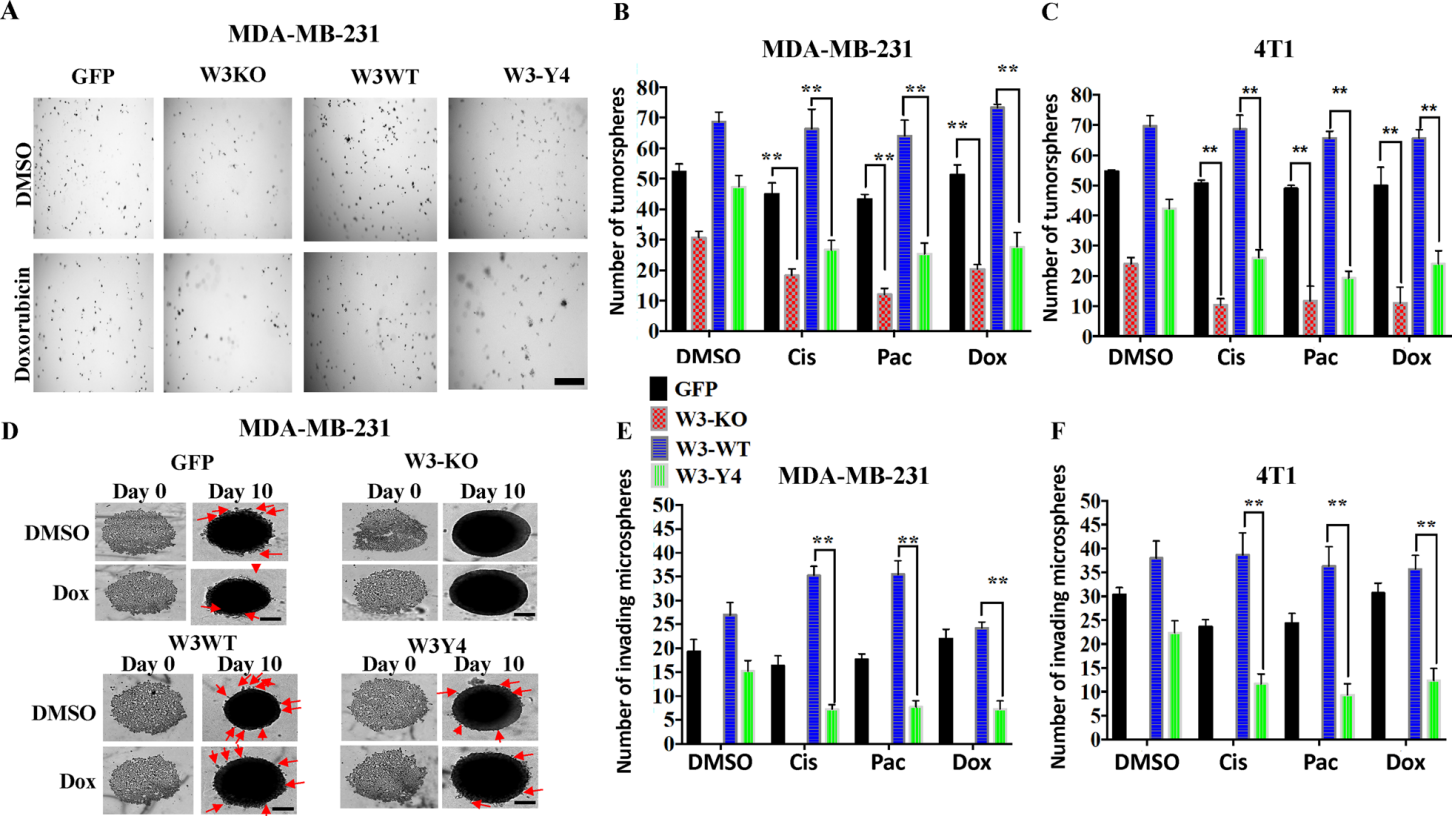
Loss of WAVE3 expression or its phosphorylation in combination with chemotherapeutics inhibits tumorsphere formation and invasion in vitro. **A** Representative micrographs of tumorspheres derived from parental MDA-MB-231 cell line and its derivatives treated with or without doxorubicin (100 nM) for 14 days. **B** and **C** Quantification of the number of tumorspheres formed with or without treatment with cisplatin (500 nM), paclitaxel (100 nM) or doxorubicin (100 nM) of MDA-MB-231 (**B**) and 4T1 cells (**C**) and their derivatives. **D** Representative micrographs of tumorsphere invasion (red arrows point to invasive microspheres) derived from parental MDA-MB-231 cells and its derivatives treated with or without doxorubicin (100 nM) for 10 days. At day 3, Matrigel was added to the tumorsphere cultures and cells that invaded the Matrigel (arrows) were recorded and quantified at day 10. **E** and **F** Quantification of the number of invasive microspheres with or without treatment with cisplatin (500 nM), paclitaxel (100 nM) or doxorubicin (100 nM) of MDA-MB-231 (**E**) and 4T1 (**F**) and their derivatives. Data are the mean ± SD (*n* = 3, **p < 0.01, Student’s t test). Data shown is representative of three independent experiments

Next, we assessed the effect of loss of WAVE3 expression and its phosphorylation, in combination with chemotherapy treatment on tumorsphere growth and invasion in 3D organotypic assays. Loss of WAVE3 expression (W3-KO) in MDA-MB-231 (Fig. 2A, B) or 4T1 (Fig. 2C) resulted in a significant (*p* < 0.01) reduction in the number of tumorspheres after treatment with cisplatin, paclitaxel, or doxorubicin. Re-expression of wild-type WAVE3 (W3-WT) restored tumorsphere growth to levels compared to the control (GFP) cells, even after treatment with the chemotherapeutic drugs. By contrast, re-expression of phosphomutant (W3-Y4) failed to activate tumorsphere growth in both the presence and absence of chemotherapy treatments. Tumorsphere invasion, as measured by the number of micro-spheres that were able to invade the Matrigel, was also inhibited in MDA-MB-231-derived tumorspheres (Fig. 2D, E) and 4T1-derived tumorspheres (Fig. 2F) as a result of loss of WAVE3 expression or its phosphorylation after treatment with cisplatin, paclitaxel, or doxorubicin. Re-expression of wildtype WAVE3 restored the tumorsphere invasion potential, even in the presence of chemotherapy, while re-expression of phosphomutant WAVE3 did not.

### Loss of WAVE3 expression or its phosphorylation in combination with chemotherapy, inhibits tumor growth in vivo

**Fig. 3:**
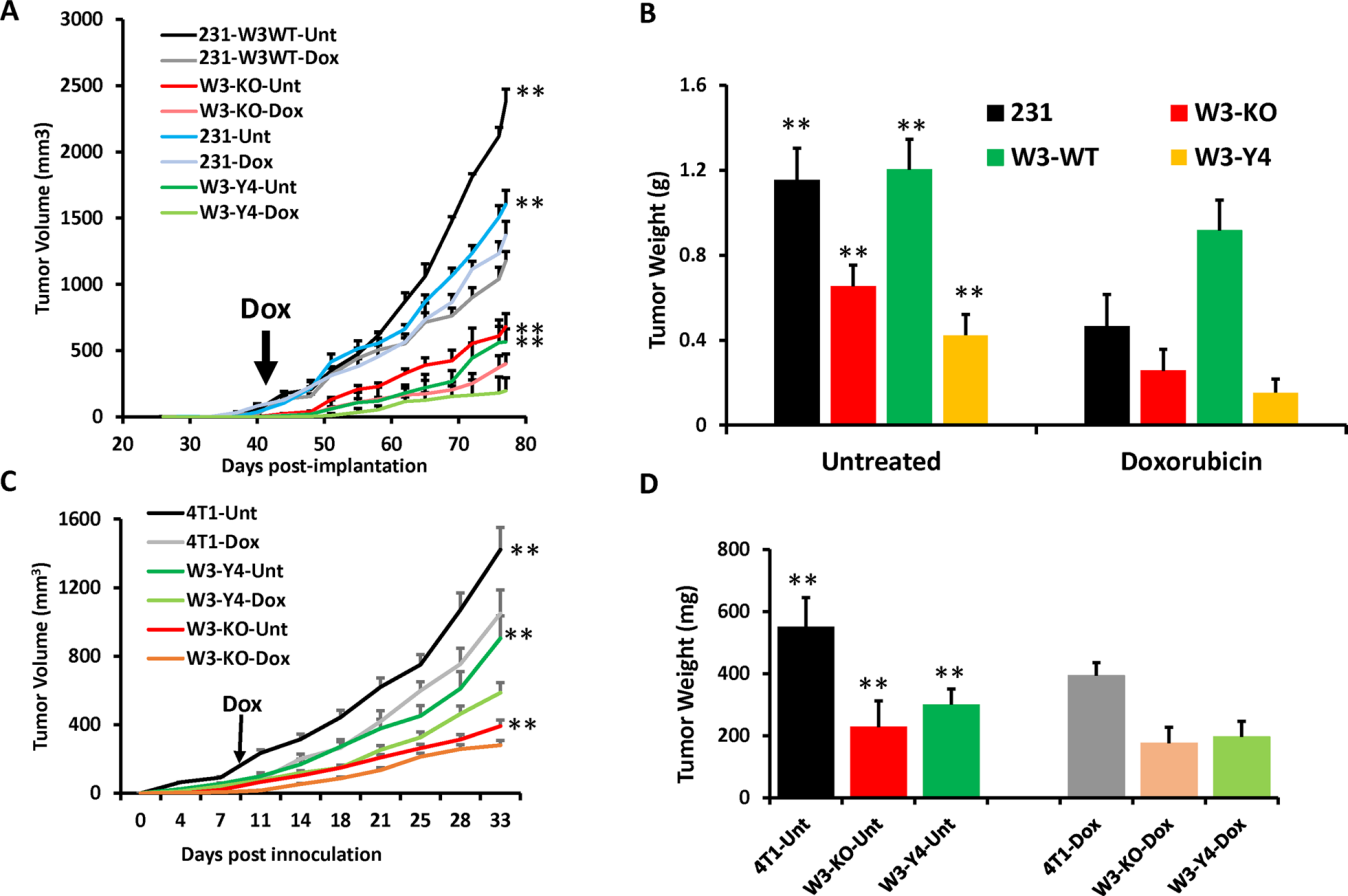
Loss of WAVE3 expression or its phosphorylation in combination with chemotherapy, inhibits tumor growth in vivo. Quantification of volume (**A**, **D**) and weight (**C**, **E**) of tumors derived from implantation of parental MDA-MB-231 (**A**, **C**) and 4T1 cells (**C**, **E**) and their derivatives into the mammary fat pads of NSG mice for MDAMB-231 and Balb/c mice for 4T1 cells with or without treatment with doxorubicin (1 mg/kg). The vertical dark arrow indicates the initiation of the treatment. Data are generated from five mice per group and each mouse was injected in both the left and right mammary fat pad, resulting in ten tumors. Data are the mean ± SD (*n* = 10, **p < 0.01; Student’s t test). Comparisons and statistical analyses (**) were done between the untreated and the treated groups

Our in vitro analyses showed that loss of WAVE3 expression or phosphorylation in combination with chemotherapy has a dramatic inhibitory effect on the oncogenic behavior of TNBC cells in vitro. Next, we tested the effect of doxorubicin, in combination with loss of WAVE3 expression or its phosphorylation in vivo. Control (231) MDA-MB-231 cells, their WAVE3-deficient (W3-KO), W3-KO re-expressing wildtype (W3-WT) or W3-KO re-expressing phosphomutant WAVE3 (W3-Y4) derivatives were injected in the mammary fat pads (fourth inguinal glands on both sides) of NSG mice (*n* = 5 mice, 10 tumors), 41 days post-injection, when the first tumors reached ~ 100 mm^3^, mice started receiving doxorubicin (Dox) or DMSO (Unt) injections twice a week for five more weeks, after which mice in all 8 groups were sacrificed and tumors harvested. In the untreated groups (Unt), mice injected with WAVE3-deficient MDA-MB-231 cells (W3-KO-Unt) developed tumors that were significantly (*p* < 0.01) smaller (Fig. 3A) and lighter (Fig. 3B) than those from mice injected with the parental (231-Unt) cells. On the other hand, mice injected with W3-KO MDA-MB-231 re-expressing wildtype WAVE3 (231-W3WT-Unt), tumor growth was not only restored, by exceeded that of the parental (231-Unt). Re-expression of phosphomutant WAVE3 (W3-Y4-Unt), however, failed to restore tumor growth. The in vivo behavior of these tumor cells is consistent with the one they showed in vitro (Figs. 1 and 2). These results are also consistent with our previously published studies [17, 18]. Contrary to the untreated groups (Unt), in the mice treated with doxorubicin (Dox), tumor growth was consistently significantly (*p* < 0.01) inhibited in all treated four groups (231-Dox, W3-KO-Dox, W3WT-Dox and W3-Y4-Dox) compared to their untreated counterparts (Fig. 3A, B). What is noteworthy is that in the mice injected with the WAVE3-deficient (W3-KO) or those re-expressing phosphomutant WAVE3 (W3-Y4), tumors that grew in the doxorubicin-treated group were significantly (*p* < 0.01) smaller (Fig. 3A) and lighter (Fig. 3B) than those that grew in their respective untreated groups, which strongly suggests a synergistic effect between loss of WAVE3 or its phosphorylation and doxorubicin treatment to inhibit tumor growth. These finding were replicated with the 4T1 cells (Fig. 3C, D). Thus, we show that loss of WAVE3 expression or its phosphorylation, in combination with chemotherapy, has a deleterious effect on BC tumor growth.

### Chronic exposure of BC cells to chemotherapy induces WAVE3 expression and activates the CSC phenotype that are associated with chemoresistance

**Fig. 4:**
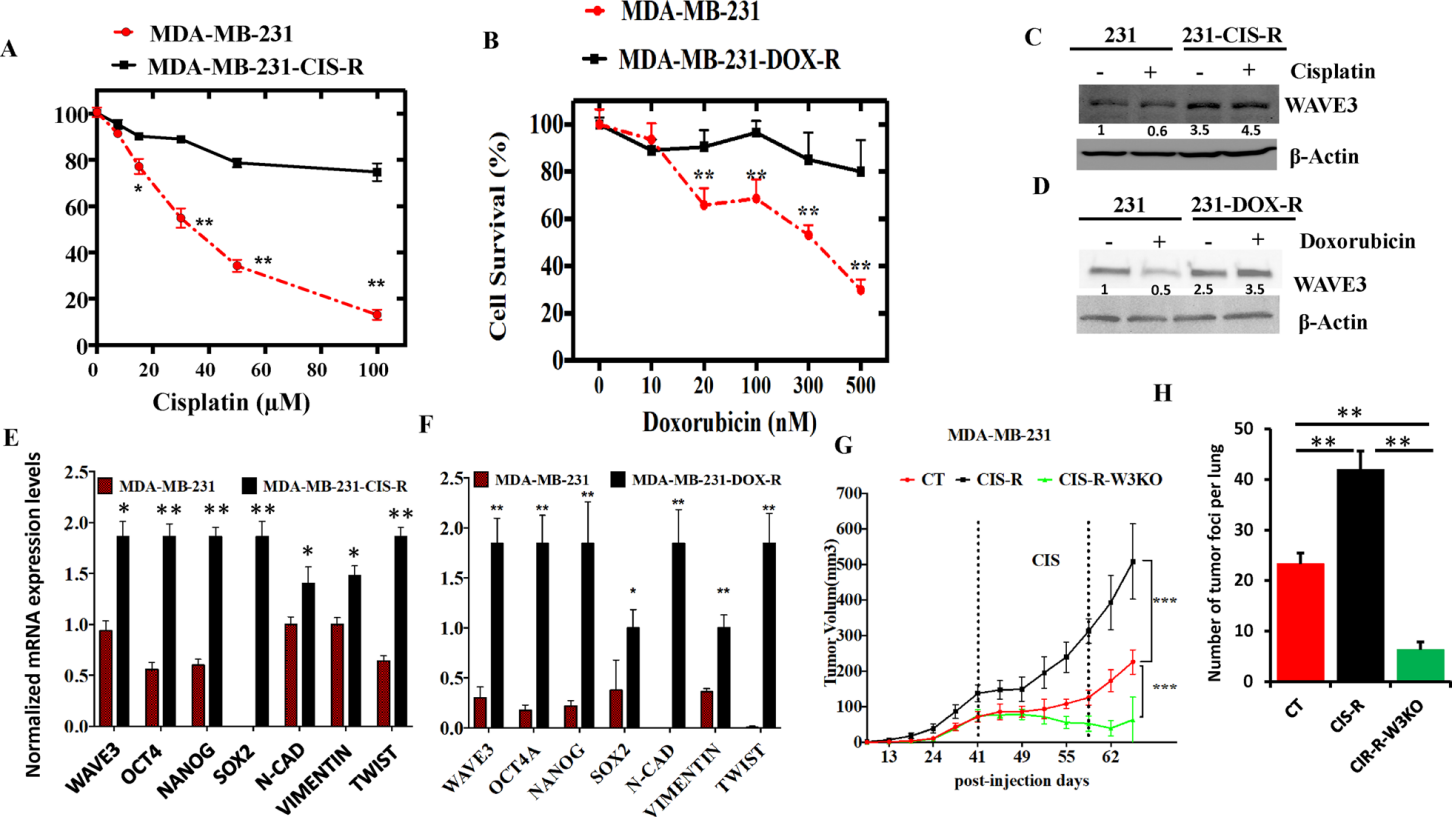
Chronic exposure of TNBC cells to chemotherapy induces WAVE3 expression and activates the CSC phenotype that are associated with chemoresistance. **A** & **B** Parental and cisplatin-resistant CIS-R (**A**) or doxorubicin-resistant DOX-R (**B**) MDA-MB-231 cells were treated with increased concentrations of cisplatin, ranging from 0 to 100 µM (Cis) and from 0 to 500 nM (Dox) for 2 days followed by MTT assay to assess for cell viability. Data are the mean ± SD (*n* = 3; **p < 0.01, Student’s t test). **C** & **D** Representative Western blot of protein lysates from parental and cisplatin-resistant (**C**) or doxorubicin-resistant (**D**) MDA-MB-231 cells that were treated with or without cisplatin (30 µM) or doxorubicin (300 nM) for 2 days and subjected to immunoblotting with antibodies against WAVE3. β-Actin was used as loading control. **E** & **F** Quantitative RT-PCR of mRNA of the indicated genes from total RNA isolated from parental and CIS-R (**E**) or DOX-R (**F**) MDA-MB-231 cells. Data are the mean ± SD (*n* = 3, *p < 0.05; **p < 0.01, Student’s *t* test). **G** Quantification of volume of tumors derived from inoculation of parental (CT), cisplatin-resistant (CIS-R) or WAVE3-deficient CIS-R (CIS-R-W3KO) MDA-MB-231 cells with treatment with cisplatin (1 mg/kg). The vertical dotted lines indicate the treatment period with cisplatin. Data are generated from five mice per group and each mouse was injected in both the left and right mammary fat pad, resulting in ten tumors. Data are the mean ± SD (*n* = 10, *p < 0.05; Student’s *t* test). **H** Quantification of lung metastatic foci from the animal experiment described in (**G**). Data are the mean ± SD (*n* = 5, **p < 0.01; Student’s t test)

Our published studies have established a link between WAVE3 expression and the acquisition of the chemotherapy resistance phenotype in TNBC tumors [12]. In addition, the data presented in Fig. 3 show that loss of WAVE3 sensitizes TNBC cells and tumors to chemotherapy. To further investigate the role of WAVE3 in the resistance to chemotherapy, we generated an MDA-MB-231 line that is resistant to Cisplatin (Fig. 4A) and Doxorubicin (Fig. 4B). The Cisplatin-resistant line (MDA-MB-231-CIS-R) showed more than 80% survival rate with Cisplatin concentrations beyond 100 µM, while most of the parental MDA-MB-231 cells were dead at this concentration (Fig. 4A). Similarly, the Doxorubicin-resistant line (MDA-MB-231-DOX-R) showed more than 90% survival rate with Doxorubicin concentrations beyond 500 nM, while most (more than 80%) of the parental MDA-MB-231 cells were dead at this concentration (Fig. 4B). Our published studies have also established an association between the WAVE3/chemoresistance axis and the cancer stem cell (CSC) phenotype [12, 18], as well as the EMT program [18]. The Cisplatin-resistant (Fig. 4C) and the Doxorubicin-resistant (Fig. 4D) MDA-MB-231 cells showed increased expression levels of WAVE3 at the protein levels, while quantitative RT-PCR showed a significant (*p* < 0.05) increase in expression levels of WAVE3 mRNA levels in the Cisplatin-resistant cells (Fig. 4E) and the Doxorubicin-resistant (Fig. 4F) MDA-MB-231 cells. CSC markers Oct4, Nanog and Sox2, as well EMT markers *N*-Cadherin, Vimentin and Twist, were also significantly (*p* < 0.05) induced in cisplatin-resistant (Fig. 4E) and the Doxorubicin-resistant MDA-MB-231 cells (Fig. 4F), which is consistent with our previously published data [12, 18]. These data show that the acquisition of the resistant phenotype of MDA-MB-231 cells is concomitant to the induction of WAVE3 expression, and suggest a positive feedback loop between WAVE3 expression and the chemoresistance phenotype, i.e., increased WAVE3 expression levels drive the chemoresistance phenotype, while the chemotherapy treatment feeds back and induces more WAVE3 expression, and the vicious cycle continues. To delve deeper into the relationship between WAVE3 and the chemoresistance phenotype, we devised an in vivo tumor growth experiment, where the parental (CT), cisplatin-resistant (CIS-R) or the cisplatin-resistant MDA-MB-231 cells that were rendered deficient in WAVE3 (CIS-R-W3KO) were orthotopically injected in the mammary fat pads of female NSG mice. Forty-one days post-tumor cells injections, when the first tumors reached an average volume of ~ 150 mm^3^, mice in all three groups were treated with cisplatin twice a week for 4 weeks, and tumor growth was followed over a total of 70 days since the first day of injection (Fig. 4G). Tumor growth was significantly (*p* < 0.001) faster, and tumors were also significantly larger in mice injected with the cisplatin-resistant MDA-MB-231 cells than their parental control counterparts. Treatment with cisplatin seemed to have slowed tumor growth in both groups during the first week of the treatment with cisplatin. However, this inhibitory effect did not last for more than a week and tumors started growing faster afterwards with a faster rate for the CIS-R tumors than the parental control (Fig. 4G; compare black and red graphs). Interestingly, when WAVE3 was knocked out in the cisplatin-resistant cells, tumor growth initially remained similar to that of the prenatal (CT) during the first ~ 40 days. However, after the chemotherapy treatment started, the CIS-R-W3KO-derived tumors started regressing and continued to regress during the entire treatment period (Fig. 4D, green curve). The resulting tumors were significantly (*p* < 0.001) smaller than both those derived from the parental or the cisplatin-resistant MDA-MB-231 cells. Tumor metastasis to the lungs was also significantly (*p* < 0.001) blunted in mice injected with the WAVE3-deficient-cisplatin-resistant cells (Fig. 4H, Additional file 1: Fig. S2). Together, these data support the notion that while increased WAVE3 expression endows cancer cells with increased chemoresistance, and increased tumorigenicity and metastasis potentials, inhibition of expression of WAVE3, on the other hand, sensitizes these cancer cells to chemotherapy, resulting in inhibition of tumor growth and metastasis.

### Loss of WAVE3 expression or phosphorylation in combination with chemotherapy induce β-Catenin degradation

**Fig. 5:**
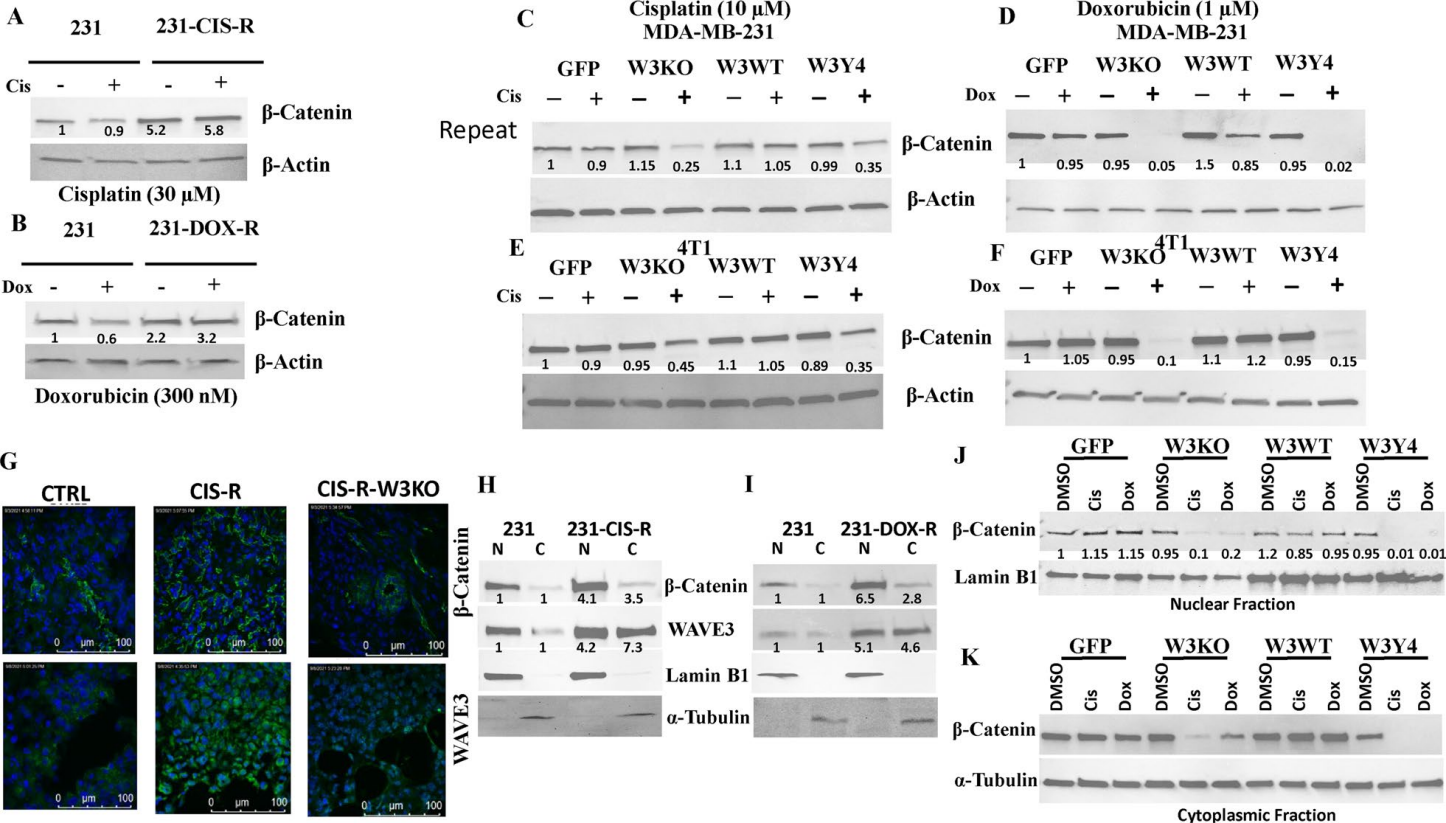
Loss of WAVE3 expression or phosphorylation in combination with chemotherapy induce β-catenin degradation. **A** & **B** Representative Western blots of protein lysates from parental and CIS-R (**A**) or DOX-R (**B**) MDA-MB-231 cells that were treated with or without cisplatin (30 µM) for 24 h, and subjected to immunoblotting with antibodies against β-Catenin. β-Actin was used as loading control. **C**–**F** Representative Western blots of protein lysates from MDA-MB-231 (**C** & **D**) or 4T1 cells (**E** & **F**) and their derivatives that were treated with or without cisplatin (**C** & **E**) or doxorubicin (**D** & **F**) for 24 h, and were subjected to immunoblotting with antibodies against β-Catenin. β-Actin was used as loading control. **G** Representative confocal microscopic images of sections of tumors derived from mice injected with parental (CTRL), cisplatin resistance (CIS-R) or WAVE3-deficient CIS-R (CIS-R-W3KO) MDA-MB-231, and stained for β-catenin (upper panels, green) or WAVE3 (lower panels, green). Cell nuclei were counterstained with DAPI (blue). Scale bar, 100 µm. **H** and **I** Representative Western blots of nuclear (*N*) or cytoplasmic (C) fraction of protein lysates from parental and CIS-R (**H**) or DOX-R (**I**) MDA-MB-231 cells that were subjected to immunoblotting with antibodies against β-catenin or WAVE3. **J** & **K** Representative Western blots of protein lysates from parental MDA-MB-231 cells and their derivatives that were treated with or without cisplatin or doxorubicin for 24 h, and were subjected to immunoblotting with antibodies against β-catenin. The nuclear fraction is analyzed in (**J**), while the cytoplasmic fraction is analyzed in (**K**). α-Tubulin and Lamin B1 were used as loading controls for the nuclear and the cytoplasmic fraction, respectively. Data shown are representative of 3 replicates. The numbers under each WB band represent the fold change in signal intensity with respect to its respective control band in each panel after normalization to the loading control signal

β-Catenin is known to be induced during EMT [21], and our published studies have established WAVE3 as a major regulator of the EMT program [22, 23], as well as plays a key role in the regulation of the chemoresistance phenotype [12]. The involvement of β-catenin signaling pathway in the regulation of resistance to chemotherapy is also well documented [24–32]. The molecular mechanisms that govern the WAVE3-mediated regulation of β-catenin function in chemoresistance have however not been fully investigated. We found expression levels of β-catenin more than quadrupled in the cisplatin-resistant MDA-MB-231 (231-CIS-R) cells as compared to their parental counterparts (Fig. 5A). Similarly, expression levels of β-catenin increased by more than threefold in the Doxorubicin-resistant MDA-MB-231 (231-DOX-R) cells (Fig. 5B). Treatment of parental MDA-MB-231 (Fig. 5C, D) or 4T1 (Fig. 5E, F) cells with either cisplatin or doxorubicin, respectively, had no noticeable effect on β-catenin expression. However, loss of WAVE3 expression (W3KO) or phosphorylation (W3KOY4), in combination with either cisplatin or doxorubicin, almost completely inhibited expression of β-catenin in both MDA-MB-MB-231 (Fig. 5C, D) and 4T1 (Fig. 5E, F). Conversely, overexpression of wildtype WAVE3 (W3WT) negated the inhibitory effects of cisplatin and doxorubicin on β-catenin expression. These finding were also confirmed in vivo by immunostaining of β-catenin in the tumors derived from the experiment described in Fig. 4 (Fig. 5G). β-Catenin (Fig. 5G; upper-middle panel) and WAVE3 (Fig. 5G; lower-middle panel) staining levels were higher in the cisplatin-resistant (CIS-R), as compared to the tumors derived from the parental (CTRL) MDA-MB-231 cells. Knockdown of WAVE3 in the cisplatin-resistant cells blunted staining of both WAVE3 (Fig. 5G; upper-right panel) and β-catenin (Fig. 5G; lower-right panel). These results suggest that WAVE3 exerts a protective effect against the chemotherapy-mediated degradation of β-catenin, and that not only expression of WAVE3, but the maintenance of WAVE3 phosphorylation is also required for this protective effect.

Given the tight link between the translocation of β-catenin to the nucleus and activation of its oncogenic pathways, we sought to determine whether the increase of β-catenin protein levels in the chemo-resistant BC cells was indeed associated with its nuclear accumulation. We used Western Blot analyses to assess the amounts of β-catenin in the nuclear fractions of parental MDA-MB-231 and its CIS-R (Fig. 5H) and DOX-R derivatives (Fig. 5I). Nuclear (N) β-catenin increased by more than fourfold in the CIS-R (Fig. 5H) and more than sixfold in the DOX-R cells (Fig. 5I), when compared to the parental MDA-MB-231 cells. Cytoplasmic (C) levels of β-catenin also increased in both the CIS-R and DOX-R cells. Similar pattern was observed for both cytoplasmic and nuclear WAVE3, that showed a significant increase in both the CIS-R (Fig. 5H) and the DOSX-R (Fig. 5I), compared to the parental cells. These results show that the acquisition of the therapy resistant phenotype (CIS-R or DOX-R) is concomitant with the accumulation of β-catenin in the nucleus, and therefore, the oncogenic behavior of the TNBC cells. Next, we assessed for the effect of loss of WAVE3 expression or phosphorylation on nuclear accumulation of β-Catenin. We found treatment of parental MDA-MB-231 with either cisplatin or doxorubicin had no noticeable effect on the amount of nuclear β-catenin (Fig. 5J). However, loss of WAVE3 expression (W3KO) or phosphorylation (W3KOY4), in combination with either cisplatin or doxorubicin, almost completely depleted nuclear β-catenin (Fig. 5J). Conversely, overexpression of wildtype WAVE3 (W3WT) negated the inhibitory effects of cisplatin and doxorubicin on nuclear accumulation of β-catenin (Fig. 5J). Similar pattern was observed for cytoplasmic β-catenin (Fig. 5K). These findings further support the WAVE3-chemotherapy combination-mediated activation of the oncogenic activity of β-catenin by inducing its nuclear translocation. Lastly, we assessed the effect of transient treatment of the prenatal MDA-MB-231 cells, their W3KO, W3WT or W3Y4 derivatives with either Cisplatin or Doxorubicin, on mRNA levels of CSC markers Oct4 and Nanog or EMT markers E-Cadherin and Vimentin. As expected and based on our previously published findings [14], mRNA expression levels of CSC markers Nanog and Oct4, and EMT marker Vimentin were significantly reduced in the W3KO cells, while those of E-Cadherin were significantly increased (Additional file 1: Fig. S3). Overexpression of W3WT restored expression of Nanog, Oct4 and Vimentin, and reduced expression of E-Cadherin, while W3Y4 did not (Additional file 1: Fig. S3). However, increased mRNA levels of these markers were not as significant as those observed in the established Cisplatin-resistant (CIS-R) or the Doxorubicin-resistant (DOX-R) MDA-MB-231 cells (Fig. 4E, F).

### Inhibition of the proteasome-induced protein degradation stabilizes β-catenin in the chemotherapy-treated WAVE3-deficient cells

**Fig. 6:**
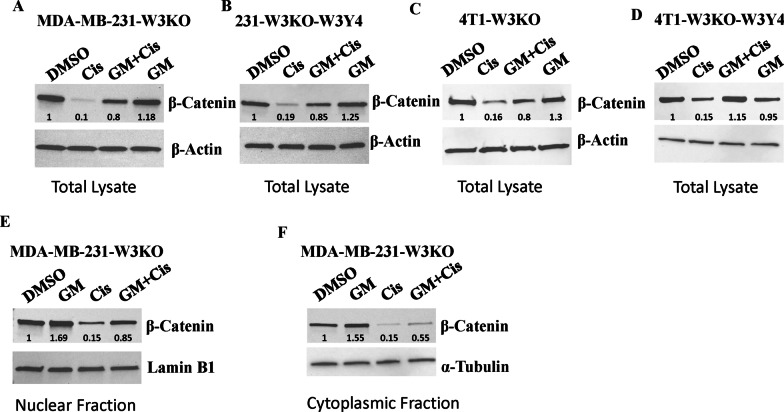
Inhibition of the proteasome-induced protein degradation stabilizes β-catenin in chemotherapy-treated WAVE3-deficient cells. **A**–**D** Representative Western blots of protein lysates from WAVE3-deficient MDA-MB-231 (MDA-MB-231-W3KO) (**A**), WAVE3-deficient 231 cells overexpressing phospho-mutant WAVE3 (231-W3KO-W3Y4) (**B**), WAVE3-deficient 4T1 (4T1-W3KO) or WAVE3-deficient and (**C**) or WAVE3-deficient 4T1 cells overexpressing phospho-mutant WAVE3 (4T1-W3KO-W3Y4) cells (**D**) that were treated for 24 h with 0.1% DMSO, 10 µM cisplatin (Cis), 1 µM GM6001 (GM), or both GM6001 and cisplatin (GM + Cis), and were subjected to immunoblotting with antibodies against β-Catenin. β-Actin was used as loading control. **E** & **F** Representative Western blots of nuclear (**E**) or cytoplasmic (**F**) fraction of protein lysates from MDA-MB-231-W3KO cells that were treated for 24 h with 0.1% DMSO, 10 µM cisplatin (Cis), 1 µM GM6001 (GM), or both GM6001 and cisplatin (GM + Cis), were subjected to immunoblotting with antibodies against β-Catenin. α-Tubulin and Lamin B1 were used as loading controls for the nuclear and the cytoplasmic fraction, respectively. The numbers under each WB band represent the fold change in signal intensity with respect to its respective control band in each panel after normalization to the loading control signal. Data shown are representative of 3 replicates

We assessed the expression of β-catenin mRNA levels by qt-RT-PCR and did not find any significant change of β-catenin levels as a result of loss of WAVE3 expression or phosphorylation in combination with chemotherapy treatment (not shown), which suggested to us that the chemotherapy-mediated degradation of β-catenin may be at the posttranslational level and may involve a proteasome-mediated degradation. To this end, WAVE3-deficient (W3KO) or W3KO expressing phospho-deficient (W3Y4) MDA-231 or 4T1 cells were treated with the proteasome inhibitor GM6001 (GM) in the presence or absence of cisplatin treatment (Cis), and β-catenin expression levels were assessed by Western Blot (Fig. 6). Treatment of the W3-deficient MDA-MB-231 cells (MDA-MB-231-W3KO, Fig. 6A) or W3-deficient 4T1 cells (4T1-W3KO, Fig. 6C) with cisplatin reduced β-catenin levels to almost undetectable levels in the 231 cells (Fig. 6A) and by almost 6 folds in the 4T1 cells (Fig. 6C), treatment with GM resulted in the rescue of β-catenin to almost normal levels. Similar results were found in the W3-deficient MDA-231-overexpressing phospho-deficient WAVE3 (231-W3KO-W3Y4, Fig. 6B) or W3-deficient 4T1-overexpressing phospho-deficient WAVE3 (4T1-W3KO-W3Y4, Fig. 6D). These data suggest that both WAVE3 expression and phosphorylation are required for the stabilization of β-Catenin. Next, we assessed for enrichment of β-catenin in both the cytoplasmic and nuclear fractions of the GM-treated W3-deficient MDA-MB-231 cells, in the presence and absence of cisplatin. Nuclear β-catenin expression levels were increased by ~ 70% in the GM-treated W3KO cells as compared to the DMSO-treated cells (Fig. 6E). While treatment with cisplatin reduced nuclear levels of β-catenin by  sixfold, GM treatment restored nuclear β-catenin to levels comparable to the DMSO-treated cells. Of note, expression levels of β-catenin in the cytoplasmic fraction followed the same trends as those of the nuclear fraction in all experimental groups (Fig. 6F). This, data further support the role of WAVE3, in the stabilization of oncogenic (nuclear) β-catenin, which drives the acquisition of the chemoresistant phenotype.

### Both β-catenin and WAVE3 expression levels correlate with the aggressiveness of breast cancer and with poor outcome in BC patients

**Fig. 7:**
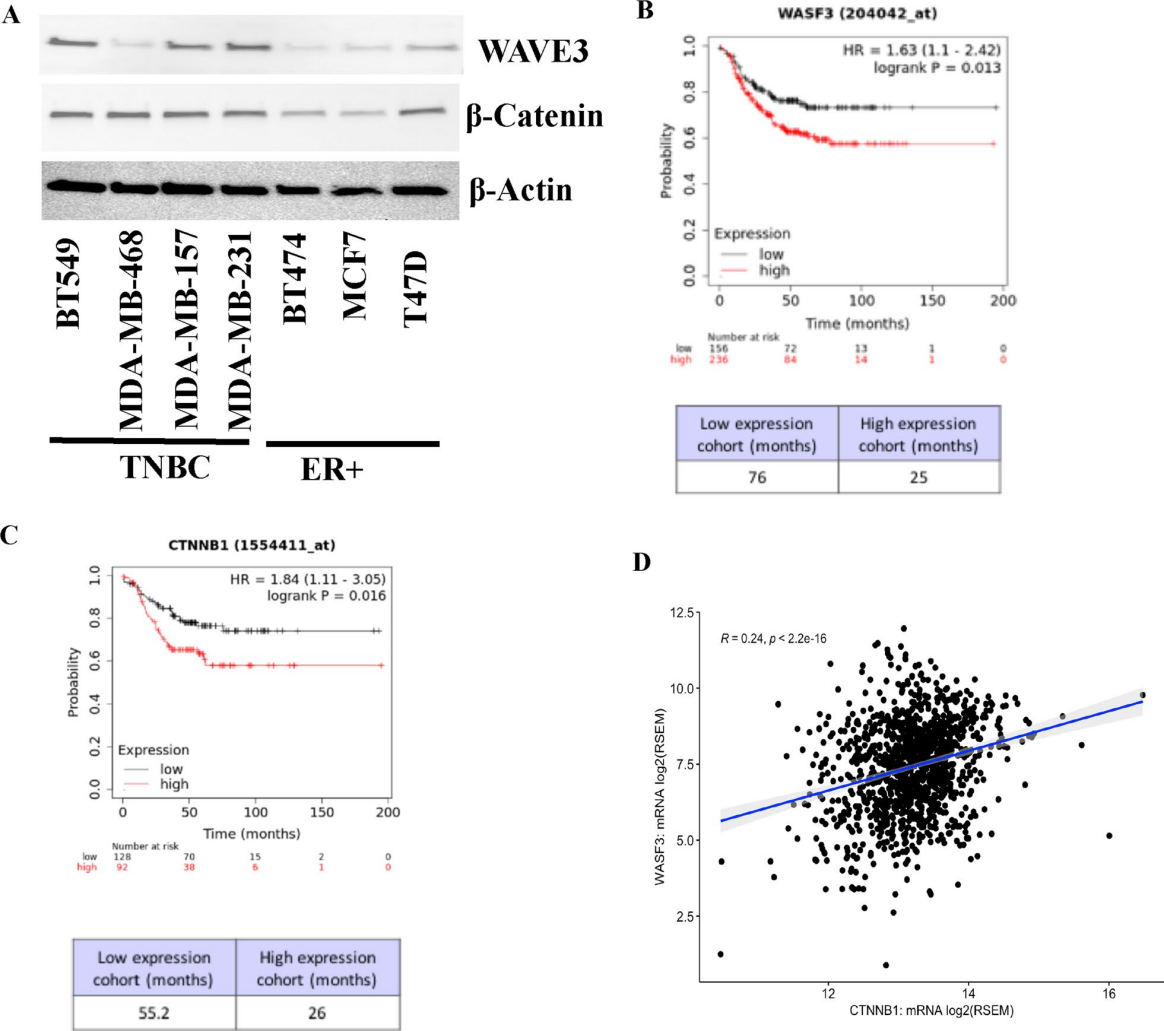
Both β-catenin and WAVE3 expression levels correlate with the aggressiveness of breast cancer and with poor outcome in BC patients. **A** Representative Western blots of protein lysates from the indicated breast cancer cell lines that were subjected to immunoblotting with antibodies against WAVE3 (upper panel) or β-catenin (middle panel). β-Actin (lower panel) was used as loading control. Data shown are representative of 3 replicates. **B**, **C** Breast Cancer Kaplan–Meier plotter (KM, http://kmplot.com/analysis/) correlating survival of triple negative breast cancer patients with expression levels of WASF3/WAVE3 (**B**) and CTNNB1/β-catenin (**C**). High WAVE3 and β-catenin expression levels correlate with poor survival probability in BC patients. **D** Interrogation of the TCGA breast cancer PanCancer Atlas cohort (*n* = 803 BC patients) showed a significant (*p* < 2.2e-16) positive correlation between WAVE3 and β-catenin mRNA expression levels

Our previously published studies established a clear association between WAVE3 and the aggressiveness with BC [20]. Western Blot analyses showed expression levels of both WAVE3 and β-catenin to be higher in the basal (TNBC) subtype BC compared to their luminal (ER+) counterparts (Fig. 7A). Furthermore, in silico analyses of the Kaplan–Meier (KM)-plotter BC datasets (*n* = 4929 BC patients) showed a very significant positive correlation between WAVE3 (Fig. 7B; *p* = 0.013) and β-catenin (Fig. 7C; *p* = 0.016), and patient survival probability. Elevated expression levels of WAVE3 expression reduced BC patient survival probability by more than 50 months (Fig. 5B), while increased levels of β-catenin reduced patient survival probability by almost 30 months (Fig. 7C). Finally, interrogation of the TCGA breast cancer PanCancer Atlas cohort (*n* = 803 BC patients) showed a significant (*p* < 2.2e^−16^) positive correlation between WAVE3 and β-catenin mRNA expression levels (Fig. 7D). Together, our data establish a clear association between WAVE3 expression and phosphorylation, the WAVE3-mediated stabilization of β-Catenin, and the chemoresistance phenotype of TNBC.

## Discussion

Treatment modalities for breast cancer largely depend on the molecular subtype and the presence ER, PR and Her2 receptors [5]. ER+ and PR+ breast cancer can benefit from hormonal therapy, while HER2-enriched breast cancer tumors can be treated with Her2-targeted therapy [33]. TNBC tumors, for which there are no FDA-approved targeted therapies, the only treatment options rely largely on cytotoxic chemotherapy [6]. Although TNBC patients show an initial response to the chemotherapy regimens, tumors frequently recur after the tumors develop resistance to the treatment [34]. Resistance to chemotherapy remains the main cause of disease recurrence, metastasis, and cancer-related mortality in patients with TNBC [35]. The molecular underpinnings of therapy resistance remain, however, largely unknown, as does the role of WAVE3 in mediating therapy resistance in TNBC. WAVE3, a member of Wiskott-Aldrich syndrome protein family [36], has recently drawn attention towards its role in the regulation of the invasion-metastasis cascade in TNBC downstream of the PI3K/TGF-β/EGF oncogenic signaling axis (Reviewed in [9]). Our published studies also established a key role of WAVE3 in the regulation of the CSC phenotype, which is tightly linked to chemoresistance [11, 12, 14, 18]. We also showed that the interaction between WAVE3 and the CSC-specific transcription factor YB1 was required for the nuclear translocation of YB1 and the subsequent activation of transcription of CSC-specific genes [14, 18]. Specifically, we found that phosphorylation of the WAVE3 proline-rich domain (PRD) is indispensable for the interaction between WAVE3 and YB1, and therefore, in mediating the maintenance of the CSC phenotype in TNBC [14]. These findings from our group and others [37–42], clearly established a major function of WAVE3 in the regulation of some of the mechanisms that regulate TNBC chemoresistance, i.e., the CSC phenotype. However, several other molecular mechanisms are known to be involved in mediating chemoresistance, including the β-catenin signaling [21, 43]. The potential role of WAVE3 in the β-Catenin-mediated regulation of chemoresistance has, however, not been investigated. Increased expression levels of β-catenin were found to correlate with drug resistance in breast cancer [24, 28, 30]. β-Catenin was also found to mediate self-renewal of breast CSCs [26, 29, 31]. β-Catenin signaling mediates chemoresistance by activating MDR1 expression, a member of ATP-binding cassette transporters, as the drug efflux in breast cancer [25, 27, 32].

In this study, we explored the role of WAVE3 and its phosphorylation in the acquisition of the chemoresistance phenotype of TNBC, through the stabilization of β-Catenin. We used a combination of in vitro and vivo mouse models, as well as genetic and pharmacologic manipulations to show that (i) loss of WAVE3 expression or its phosphorylation sensitizes TNBC cell lines to chemotherapeutics both in vitro in and in vivo; (ii) WAVE3 expression is increased in TNBC cells after chronic exposure to chemotherapy; the cisplatin-resistant (CIS-R) and doxorubicin-resistant (DOX-R) MDA-MB-231 cells express significantly higher levels of WAVE3 than their control counterparts, which results in increased tumor growth of the CIR-R cells; (iii) increased WAVE3 expression results in activation of β-catenin expression and its accumulation in the nucleus, while loss of WAVE3 expression and/or phosphorylation leads to decreased β-catenin protein levels and its depletion from the nucleus, even after exposure to chemotherapy; (iv) WAVE3 is required for the stabilization of β-catenin, since loss of WAVE3 expression and/or phosphorylation results in β-catenin degradation, and treatment of WAVE3-deficient or WAVE3-phospho-deficient MDA-231 cell with proteasome degradation inhibitors rescues β-catenin expression. (v) Given the association between the accumulation of β-catenin in the nucleus and its oncogenic activity, loss of WAVE3 expression or its phosphorylation inhibited β-catenin nuclear accumulation, while chronic exposure to chemotherapy in CIS-R and DOX-R cells increased nuclear β-catenin, which is concomitant to increased WAVE3 accumulation in the nucleus; and (vi) both WAVE3 and β-catenin expression levels are increased in the aggressive TNBC cells, compared to their less aggressive ER + counterparts, and increased levels of both WAVE3 and β-catenin correlate with poor disease outcomes and decreased survival probability of BC patients.

Early studies reported that WAVE3 is one of the regulators of YB1 [11], and YB1 can mediate breast cancer stem cells activity and therapy resistance [44–51]. We established a cisplatin-resistant (CIS-R) and doxorubicin-resistant (DOX-R) MDA-MB-231 cell line derivatives, and found that, in addition to increased WAVE3 and CSC markers expression levels in the CIS-R and DOX-R cells, expression levels of β-catenin were also significantly increased, which was reflected in increased tumor burden in mice injected with the CIS-R cells, even after treatment with chemotherapy. Tumors derived from CIS-R-W3KO, which also expressed significantly low levels of β-Catenin, grew much slower than their CIS-R counterparts, started regressing and continued to regress during the chemotherapy treatment, clearly establishing a key role of WAVE3/β-catenin (loss of expression of WAVE3 and β-Catenin) in sensitizing TNBC tumors to chemotherapy. Interestingly, given that we found β-catenin expression is regulated by WAVE3, with the loss of WAVE3 expression (W3-KO) or phosphorylation (W3-KO-Y4), it was not clear how this regulation takes place. One possible mechanism could be through the WAVE3-mediated stabilization β-Catenin. Indeed, on one hand, β-catenin expression levels increased in the WAVE3-expressing cells after treatment with chemotherapy, while they decrease as a result of loss of WAVE3 expression or its phosphorylation. On the other hand, however, treatment with GM6001, a proteasome degradation inhibitor can restore β-catenin expression and nuclear accumulation in the cells lacking WAVE3 expression or phosphorylation, even in the presence of chemotherapy. Other findings supporting the potential role of the WAVE3-mediated stabilization of β-catenin comes from published studies establishing the role of microRNA miR-200 in the regulation of β-catenin downstream of Zeb1 signaling axis [52]. Interestingly, our published studies have shown that WAVE3 expression is regulated by miR-200 through specific targeting of its 3′-UTR [22]. Conversely, and in a negative feedback regulatory loop, a report by Teng et al. [53], showed that WAVE3 regulates miR-200 inactivation by Zeb1 through suppression of KISS1. Another potential mechanism, whereby WAVE3 regulates β-catenin is through the WAVE3-mediated regulation of the transcription factor activity of YB1. Our published studies have shown that WAVE3 is required for YB1 nuclear translocation, where YB1 exerts its transcription factor function to activate CSC-specific genes [11, 14]. Interestingly, a recent study by Lim and colleagues [54] showed that YB1 activates the invasive potential of TNBC tumors through the regulation of expression of β-catenin. While our studies established that WAVE3 expression and phosphorylation are required for the stabilization of β-catenin, therefore, contributing to chemoresistance, more investigations are, however required to determine how WAVE3 is mediating the stabilization of β-catenin. In conclusion, we demonstrated that WAVE3 expression and phosphorylation enhance chemoresistance of TNBC cell lines and tumors in part through the WAVE3-mediated stabilization of β-catenin. These findings provide the justification for the development of novel WAVE3/β-catenin targeted therapies to alleviate chemoresistance in TNBC tumors.

## Supplementary Information

**Additional file 1**. **Fig. S1**: **A** Graphic representation of the WAVE3 functional domains and GFP-fused truncation mutants. The location of the four tyrosine residues is also shown. BR basic region, PRD proline-rich domain, VCA verprolin, cofilin, and acidic. **B** and **C** Representative Western blots of protein lysates from parental MDA-MB-231 cells (GFP), their WAVE3-deficient derivatives (W3-KO), or the W3-KO expressing either wildtype (W3-WT) or phospho-mutant (W3-Y4) WAVE3 that were subjected to immunoblotting with antibodies against WAVE3. β-Actin was used as loading control. **Fig. S2**: **A** Representative images of H&E staining of lung tissues sections of mice injected in the mammary fat pads with parental (CTRL), cisplatin resistance (CIS-R) or WAVE3-deficient CIS-R (CIS-R-W3KO) MDA-MB-231 with the treatment of cisplatin (1 mg/Kg). **B** Quantification of lung metastases from the mice described in (**A**). Data are the mean ± SD (*n* = 5, *p < 0.05; Student’s *t* test). **Fig. S3**: Quantitative RT-PCR of mRNA of the indicated genes from total RNA isolated from parental (CT), WAVE3-deficient (W3KO), W3KO-overexpressing wildtype WAVE3 ((W3WT) or phospho-mutant WAVE3 (W3Y4) MDA-MB-231 cells. Data are the mean ± SD (*n* = 3, *p < 0.05; **p < 0.01, Student’s *t* test).

**Additional file 2**: Contains uncropped Western Blots.

## Data Availability

All data generated or analyzed during this study are included in this published article and its Additional file 1, Additional file 2.

## References

[1] Siegel RL, Miller KD, Fuchs HE, Jemal A (2022). Cancer statistics, 2022. CA Cancer J Clin.

[2] Perou CM, Sorlie T, Eisen MB, van de Rijn M, Jeffrey SS, Rees CA (2000). Molecular portraits of human breast tumours. Nature.

[3] Sorlie T, Perou CM, Tibshirani R, Aas T, Geisler S, Johnsen H (2001). Gene expression patterns of breast carcinomas distinguish tumor subclasses with clinical implications. Proc Natl Acad Sci U S A.

[4] Chen X, Li J, Gray WH, Lehmann BD, Bauer JA, Shyr Y (2012). TNBCtype: a subtyping tool for triple-negative breast Cancer. Cancer Inform.

[5] Lehmann BD, Bauer JA, Chen X, Sanders ME, Chakravarthy AB, Shyr Y (2011). Identification of human triple-negative breast cancer subtypes and preclinical models for selection of targeted therapies. J Clin Invest.

[6] Foulkes WD, Smith IE, Reis-Filho JS (2010). Triple-negative breast cancer. N Engl J Med.

[7] Schneider BP, Winer EP, Foulkes WD, Garber J, Perou CM, Richardson A (2008). Triple-negative breast cancer: risk factors to potential targets. Clin Cancer Res.

[8] Sossey-Alaoui K (2013). Surfing the big WAVE: Insights into the role of WAVE3 as a driving force in cancer progression and metastasis. Semin Cell Dev Biol.

[9] Loveless R, Teng Y (2021). Targeting WASF3 signaling in metastatic cancer. Int J Mol Sci.

[10] Kansakar U, Wang W, Markovic V, Sossey-Alaoui K (2020). Elucidating the molecular signaling pathways of WAVE3. Ann Transl Med.

[11] Bledzka K, Schiemann B, Schiemann WP, Fox P, Plow EF, Sossey-Alaoui K (2017). The WAVE3-YB1 interaction regulates cancer stem cells activity in breast cancer. Oncotarget.

[12] Davuluri G, Schiemann WP, Plow EF, Sossey-Alaoui K (2014). Loss of WAVE3 sensitizes triple-negative breast cancers to chemotherapeutics by inhibiting the STAT-HIF-1alpha-mediated angiogenesis. JAKSTAT.

[13] Davuluri G, Augoff K, Schiemann WP, Plow EF, Sossey-Alaoui K (2014). WAVE3-NFkappaB interplay is essential for the survival and invasion of cancer cells. PLoS ONE.

[14] Kansakar U, Wang W, Markovic V, Sossey-Alaoui K (2021). Phosphorylation of the proline-rich domain of WAVE3 drives its oncogenic activity in breast cancer. Sci Rep.

[15] Mohammed MK, Shao C, Wang J, Wei Q, Wang X, Collier Z (2016). Wnt/beta-catenin signaling plays an ever-expanding role in stem cell self-renewal, tumorigenesis and cancer chemoresistance. Genes Dis.

[16] Qayoom H, Wani NA, Alshehri B, Mir MA (2021). An insight into the cancer stem cell survival pathways involved in chemoresistance in triple-negative breast cancer. Future Oncol.

[17] Sossey-Alaoui K, Li X, Cowell JK (2007). c-Abl-mediated phosphorylation of WAVE3 is required for lamellipodia formation and cell migration. J Biol Chem.

[18] Wang W, Kansakar U, Markovic V, Wang B, Sossey-Alaoui K (2020). WAVE3 phosphorylation regulates the interplay between PI3K, TGF-beta, and EGF signaling pathways in breast cancer. Oncogenesis.

[19] Rana PS, Wang W, Alkrekshi A, Markovic V, Khiyami A, Chan R (2021). YB1 is a major contributor to health disparities in triple negative breast cancer. Cancers (Basel).

[20] Kulkarni S, Augoff K, Rivera L, McCue B, Khoury T, Groman A (2012). Increased expression levels of WAVE3 are associated with the progression and metastasis of triple negative breast cancer. PLoS ONE.

[21] Merikhian P, Eisavand MR, Farahmand L (2021). Triple-negative breast cancer: understanding Wnt signaling in drug resistance. Cancer Cell Int.

[22] Sossey-Alaoui K, Bialkowska K, Plow EF (2009). The miR200 family of microRNAs regulates WAVE3-dependent cancer cell invasion. J Biol Chem.

[23] Taylor MA, Davuluri G, Parvani JG, Schiemann BJ, Wendt MK, Plow EF (2013). Upregulated WAVE3 expression is essential for TGF-beta-mediated EMT and metastasis of triple-negative breast cancer cells. Breast Cancer Res Treat.

[24] Braggio D, Zewdu A, Londhe P, Yu P, Lopez G, Batte K (2020). beta-catenin S45F mutation results in apoptotic resistance. Oncogene.

[25] Flahaut M, Meier R, Coulon A, Nardou KA, Niggli FK, Martinet D (2009). The Wnt receptor FZD1 mediates chemoresistance in neuroblastoma through activation of the Wnt/beta-catenin pathway. Oncogene.

[26] Krishnamurthy N, Kurzrock R (2018). Targeting the Wnt/beta-catenin pathway in cancer: update on effectors and inhibitors. Cancer Treat Rev.

[27] Liu YY, Gupta V, Patwardhan GA, Bhinge K, Zhao Y, Bao J (2010). Glucosylceramide synthase upregulates MDR1 expression in the regulation of cancer drug resistance through cSrc and beta-catenin signaling. Mol Cancer.

[28] Quinn HM, Vogel R, Popp O, Mertins P, Lan L, Messerschmidt C (2021). YAP and beta-catenin cooperate to drive oncogenesis in basal breast cancer. Cancer Res.

[29] Tang T, Guo C, Xia T, Zhang R, Zen K, Pan Y (2019). LncCCAT1 promotes breast cancer stem cell function through activating WNT/beta-catenin signaling. Theranostics.

[30] Won HS, Lee KM, Oh JE, Nam EM, Lee KE (2016). Inhibition of beta-catenin to overcome endocrine resistance in tamoxifen-resistant breast cancer cell line. PLoS ONE.

[31] Zhang F, Li P, Liu S, Yang M, Zeng S, Deng J (2021). β-Catenin-CCL2 feedback loop mediates crosstalk between cancer cells and macrophages that regulates breast cancer stem cells. Oncogene.

[32] Zhang ZM, Wu JF, Luo QC, Liu QF, Wu QW, Ye GD (2016). Pygo2 activates MDR1 expression and mediates chemoresistance in breast cancer via the Wnt/beta-catenin pathway. Oncogene.

[33] Anders CK, Carey LA (2009). Biology, metastatic patterns, and treatment of patients with triple-negative breast cancer. Clin Breast Cancer.

[34] Newton EE, Mueller LE, Treadwell SM, Morris CA, Machado HL (2022). Molecular targets of triple-negative breast cancer: where do we stand?. Cancers.

[35] Ferrari P, Scatena C, Ghilli M, Bargagna I, Lorenzini G, Nicolini A (2022). Molecular mechanisms, biomarkers and emerging therapies for chemotherapy resistant TNBC. Int J Mol Sci.

[36] Suetsugu S, Miki H, Takenawa T (1999). Identification of two human WAVE/SCAR homologues as general actin regulatory molecules which associate with the Arp2/3 complex. Biochem Biophys Res Commun.

[37] Limaye AJ, Bendzunas GN, Whittaker MK, LeClair TJ, Helton LG, Kennedy EJ (2022). In silico optimized stapled peptides targeting WASF3 in breast cancer. ACS Med Chem Lett.

[38] Teng Y, Loveless R, Benson EM, Sun L, Shull AY, Shay C (2021). SHOX2 cooperates with STAT3 to promote breast cancer metastasis through the transcriptional activation of WASF3. J Exp Clin Cancer Res.

[39] Shibuya N, Kakeji Y, Shimono Y (2020). MicroRNA-93 targets WASF3 and functions as a metastasis suppressor in breast cancer. Cancer Sci.

[40] Gou XJ, Bai HH, Liu LW, Chen HY, Shi Q, Chang LS (2020). Asiatic acid interferes with invasion and proliferation of breast cancer cells by inhibiting WAVE3 activation through PI3K/AKT signaling pathway. Biomed Res Int.

[41] Teng Y, Bahassan A, Dong D, Hanold LE, Ren X, Kennedy EJ (2016). Targeting the WASF3-CYFIP1 complex using stapled peptides suppresses cancer cell invasion. Cancer Res.

[42] Teng Y, Ross JL, Cowell JK (2014). The involvement of JAK-STAT3 in cell motility, invasion, and metastasis. JAKSTAT.

[43] Xu X, Zhang M, Xu F, Jiang S (2020). Wnt signaling in breast cancer: biological mechanisms, challenges and opportunities. Mol Cancer.

[44] Dhillon J, Astanehe A, Lee C, Fotovati A, Hu K, Dunn SE (2010). The expression of activated Y-box binding protein-1 serine 102 mediates trastuzumab resistance in breast cancer cells by increasing CD44+ cells. Oncogene.

[45] Fujita T, Ito K, Izumi H, Kimura M, Sano M, Nakagomi H (2005). Increased nuclear localization of transcription factor Y-box binding protein 1 accompanied by up-regulation of P-glycoprotein in breast cancer pretreated with paclitaxel. Clin Cancer Res.

[46] Guay D, Evoy AA, Paquet E, Garand C, Bachvarova M, Bachvarov D (2008). The strand separation and nuclease activities associated with YB-1 are dispensable for cisplatin resistance but overexpression of YB-1 in MCF7 and MDA-MB-231 breast tumor cells generates several chemoresistance signatures. Int J Biochem Cell Biol.

[47] Habibi G, Leung S, Law JH, Gelmon K, Masoudi H, Turbin D (2008). Redefining prognostic factors for breast cancer: YB-1 is a stronger predictor of relapse and disease-specific survival than estrogen receptor or HER-2 across all tumor subtypes. Breast Cancer Res.

[48] Ito T, Kamijo S, Izumi H, Kohno K, Amano J, Ito K (2012). Alteration of Y-box binding protein-1 expression modifies the response to endocrine therapy in estrogen receptor-positive breast cancer. Breast Cancer Res Treat.

[49] Janz M, Harbeck N, Dettmar P, Berger U, Schmidt A, Jurchott K (2002). Y-box factor YB-1 predicts drug resistance and patient outcome in breast cancer independent of clinically relevant tumor biologic factors HER2, uPA and PAI-1. Int J Cancer.

[50] Sutherland BW, Kucab J, Wu J, Lee C, Cheang MC, Yorida E (2005). Akt phosphorylates the Y-box binding protein 1 at Ser102 located in the cold shock domain and affects the anchorage-independent growth of breast cancer cells. Oncogene.

[51] Tiwari A, Iida M, Kosnopfel C, Abbariki M, Menegakis A, Fehrenbacher B (2020). Blocking Y-box binding protein-1 through simultaneous targeting of PI3K and MAPK in triple negative breast cancers. Cancers.

[52] Schmalhofer O, Brabletz S, Brabletz T (2009). E-cadherin, beta-catenin, and ZEB1 in malignant progression of cancer. Cancer Metastasis Rev.

[53] Teng Y, Mei Y, Hawthorn L, Cowell JK (2014). WASF3 regulates miR-200 inactivation by ZEB1 through suppression of KISS1 leading to increased invasiveness in breast cancer cells. Oncogene.

[54] Lim JP, Nair S, Shyamasundar S, Chua PJ, Muniasamy U, Matsumoto K (2019). Silencing Y-box binding protein-1 inhibits triple-negative breast cancer cell invasiveness via regulation of MMP1 and beta-catenin expression. Cancer Lett.

