# Supplementary figures and images for: The WAVE3/β-catenin oncogenic signaling regulates chemoresistance in triple negative breast cancer
Source: Breast Cancer Res. 2023 Mar 22;25:31. doi: 10.1186/s13058-023-01634-3 (PMC10035207; doi:10.1186/s13058-023-01634-3)

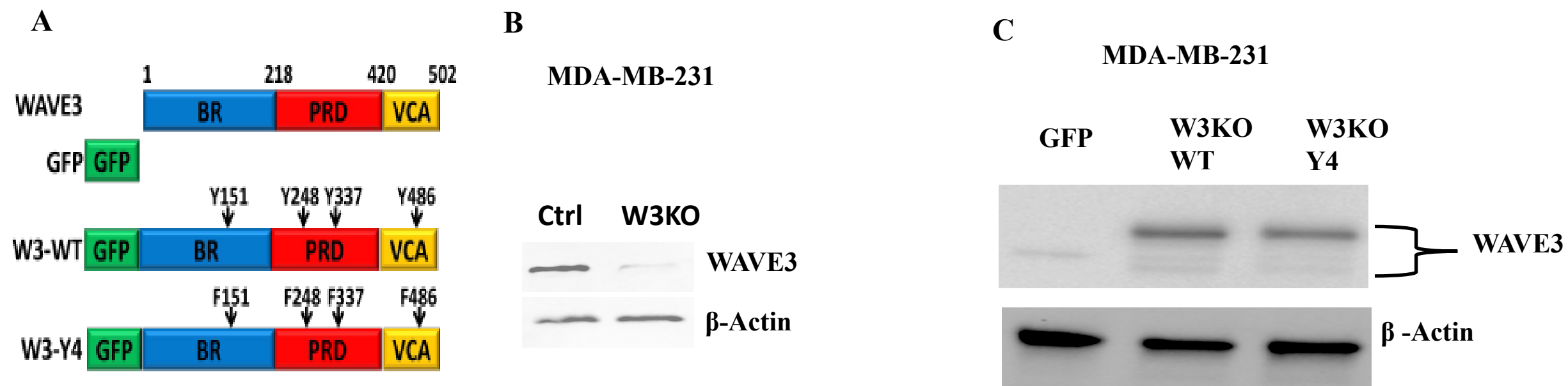

**Figure S1**

**A****CTRL****CIS-R****CIS-R-W3KO**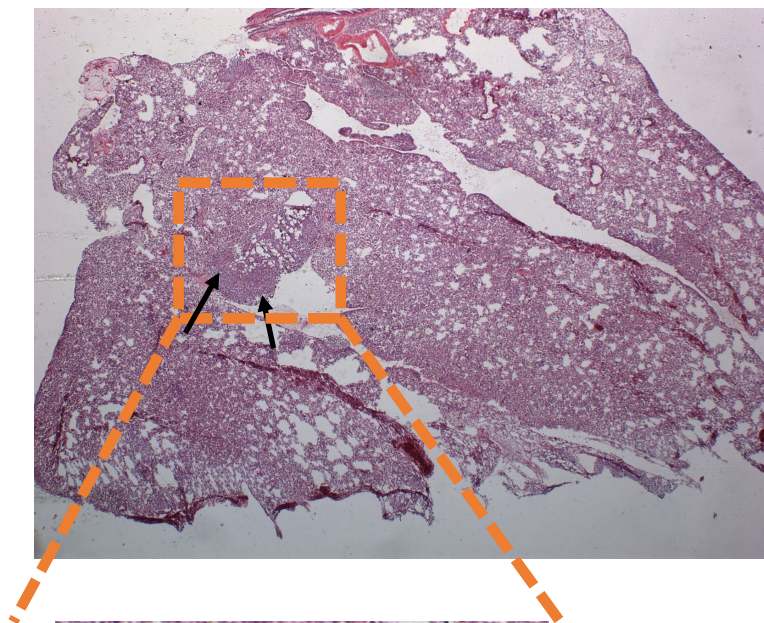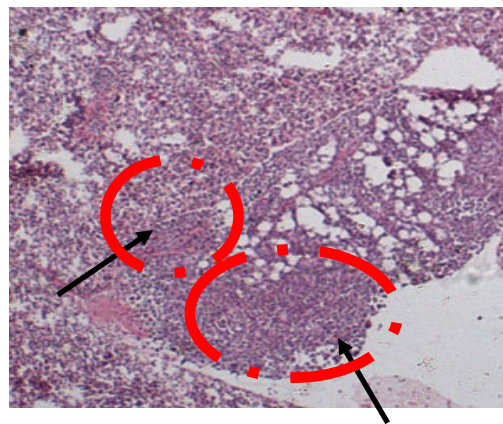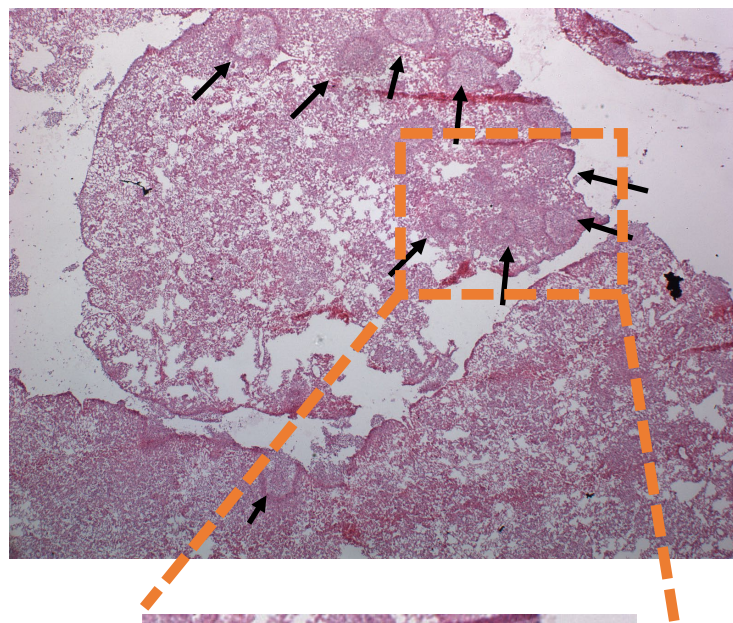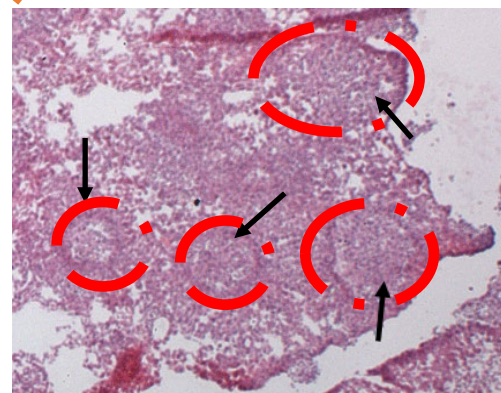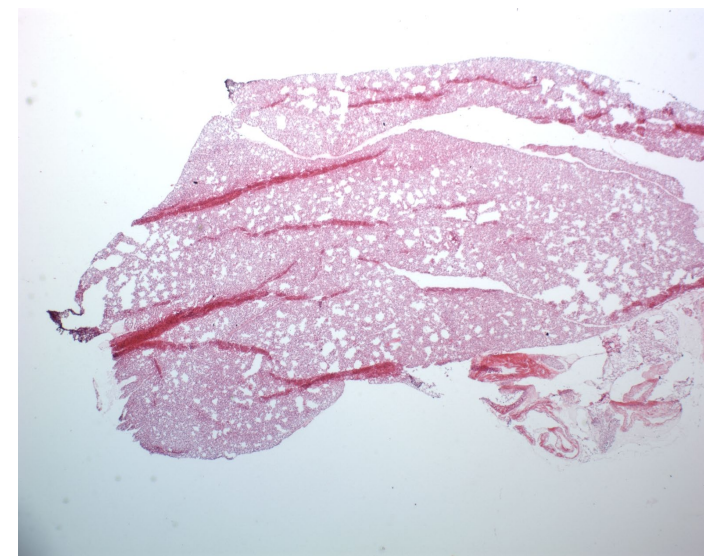**B****MDA-MB-231**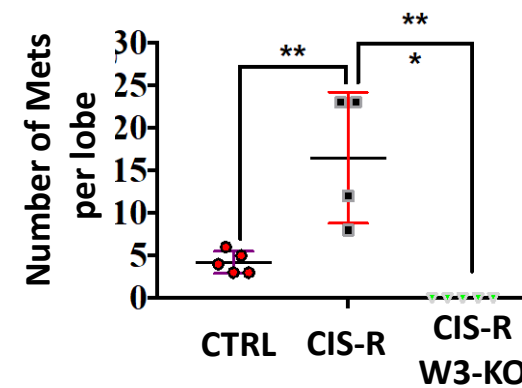**Figure S2**

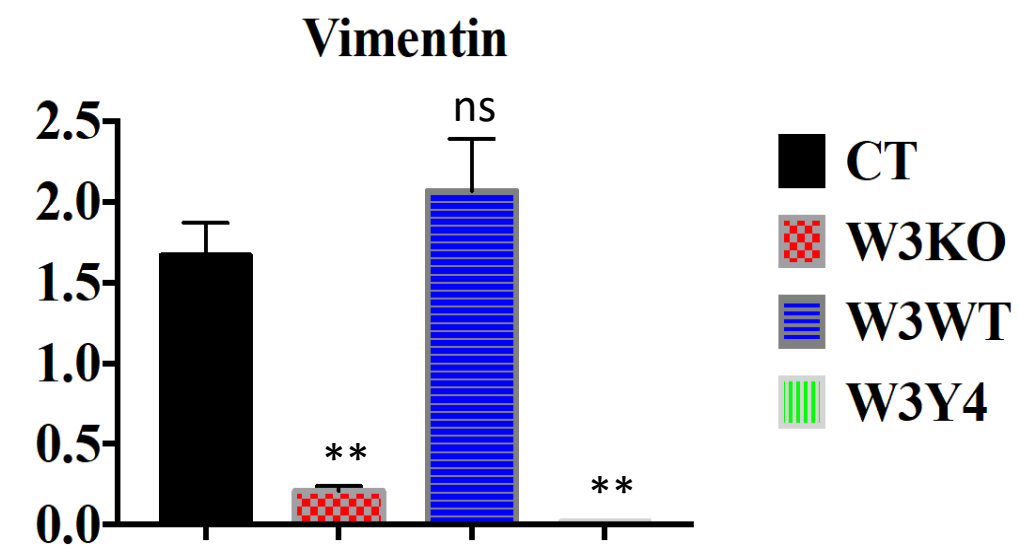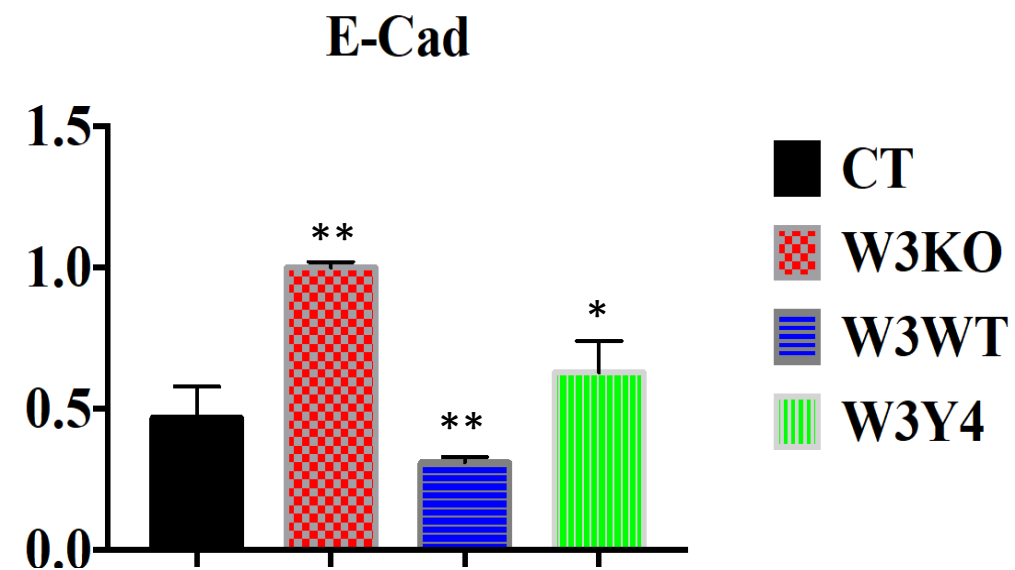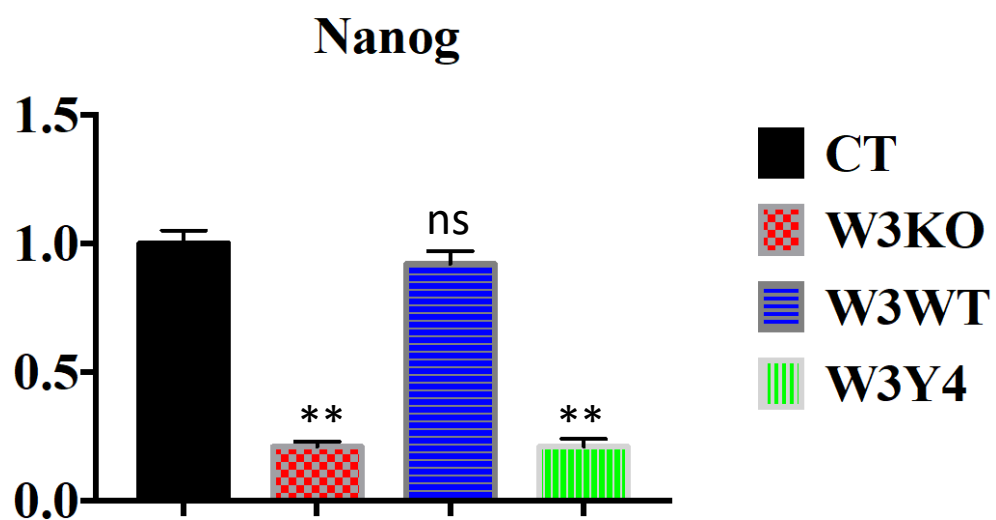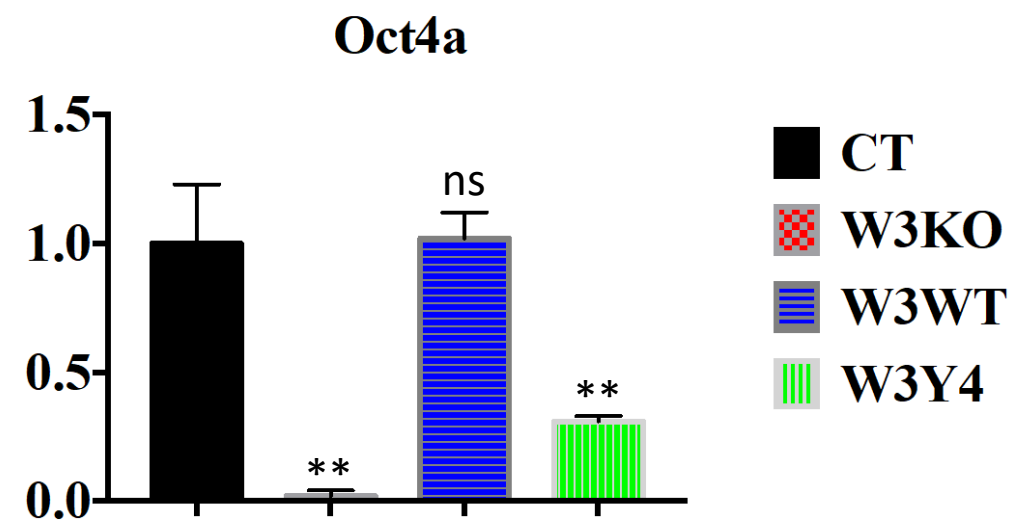

Figure S3

Supplement: Supplementary file 1 — Additional file 1. Fig. S1: A Graphic representation of the WAVE3 functional domains and GFP-fused truncation mutants. The location of the four tyrosine residues is also shown. BR basic region, PRD proline-rich domain, VCA verprolin, cofilin, and acidic. B and C Representative Western blots of protein lysates from parental MDA-MB-231 cells (GFP), their WAVE3-deficient derivatives (W3-KO), or the W3-KO expressing either wildtype (W3-WT) or phospho-mutant (W3-Y4) WAVE3 that were subjected to immunoblotting with antibodies against WAVE3. β-Actin was used as loading control. Fig. S2: A Representative images of H&E staining of lung tissues sections of mice injected in the mammary fat pads with parental (CTRL), cisplatin resistance (CIS-R) or WAVE3-deficient CIS-R (CIS-R-W3KO) MDA-MB-231 with the treatment of cisplatin (1 mg/Kg). B Quantification of lung metastases from the mice described in (A). Data are the mean ± SD (n = 5, *p < 0.05; Student’s t test). Fig. S3: Quantitative RT-PCR of mRNA of the indicated genes from total RNA isolated from parental (CT), WAVE3-deficient (W3KO), W3KO-overexpressing wildtype WAVE3 ((W3WT) or phospho-mutant WAVE3 (W3Y4) MDA-MB-231 cells. Data are the mean ± SD (n = 3, *p < 0.05; **p < 0.01, Student’s t test). [file 13058_2023_1634_MOESM1_ESM.pdf]
