# Supplementary material for: The WAVE3/β-catenin oncogenic signaling regulates chemoresistance in triple negative breast cancer
Source: Breast Cancer Res. 2023 Mar 22;25:31. doi: 10.1186/s13058-023-01634-3 (PMC10035207; doi:10.1186/s13058-023-01634-3)

Figure 4

Fig. 4C: WAVE3

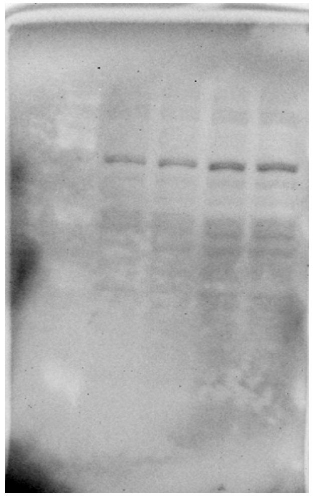

Fig. 4D: WAVE3

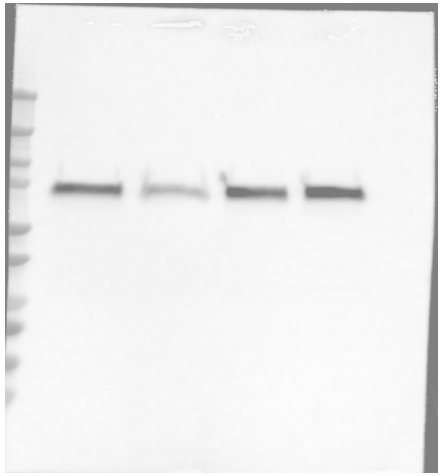

Fig. 4C: β-Actin

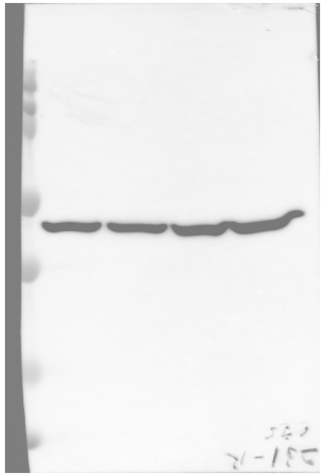

Fig. 4D: β-Actin

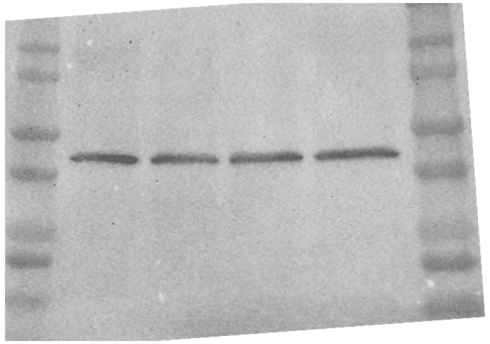

Figure 5

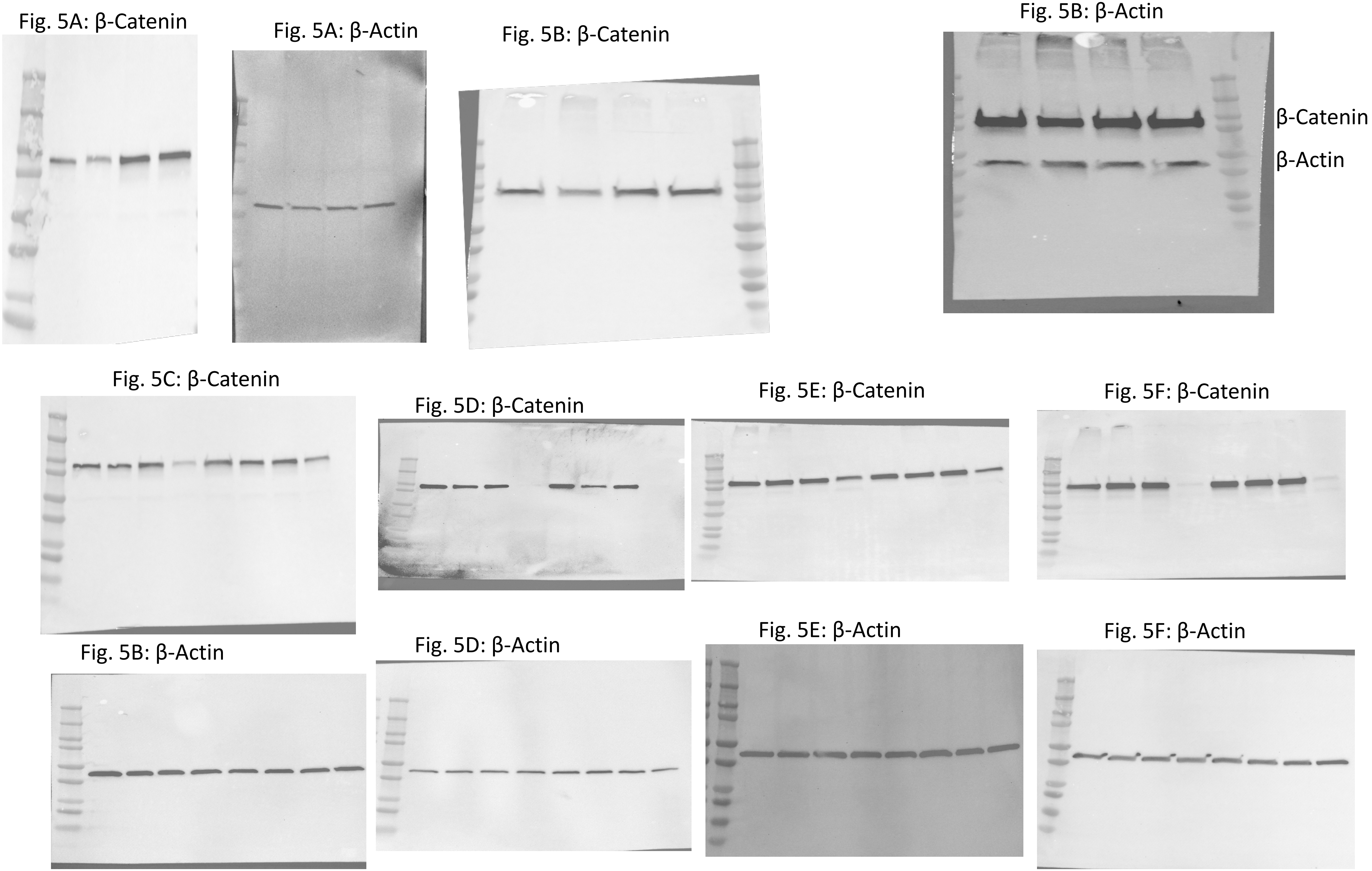
Fig. 5A: β-Catenin
Fig. 5A: β-Actin
Fig. 5B: β-Catenin
Fig. 5B: β-Actin
β-Catenin
β-Actin
Fig. 5C: β-Catenin
Fig. 5D: β-Catenin
Fig. 5E: β-Catenin
Fig. 5F: β-Catenin
Fig. 5B: β-Actin
Fig. 5D: β-Actin
Fig. 5E: β-Actin
Fig. 5F: β-Actin

# Figure 5, continued

Fig. 5H: β-Catenin

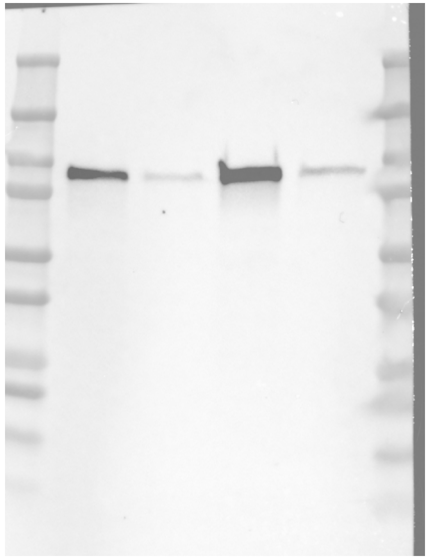

Fig. 5H: WAVE3

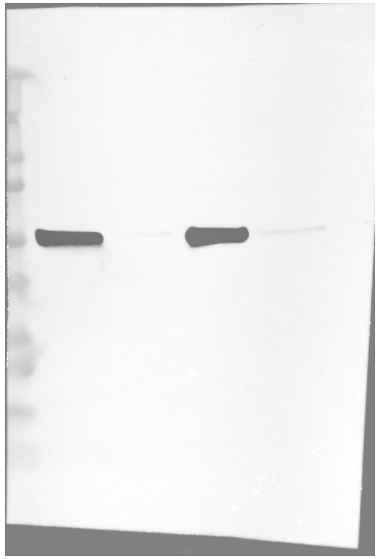

Fig. 5H: α-Tubulin

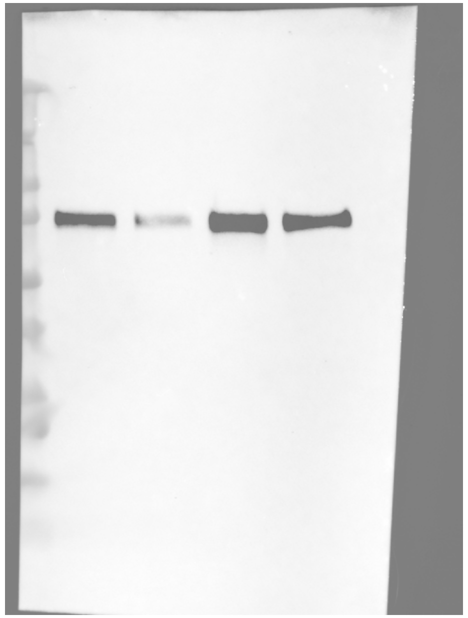

Fig. 5H: Lamin B1

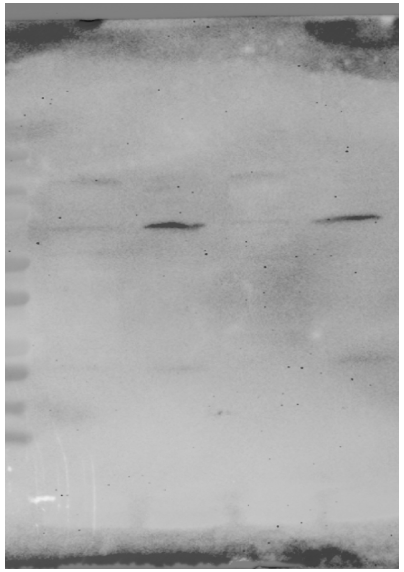

Fig. 5I: β-Catenin

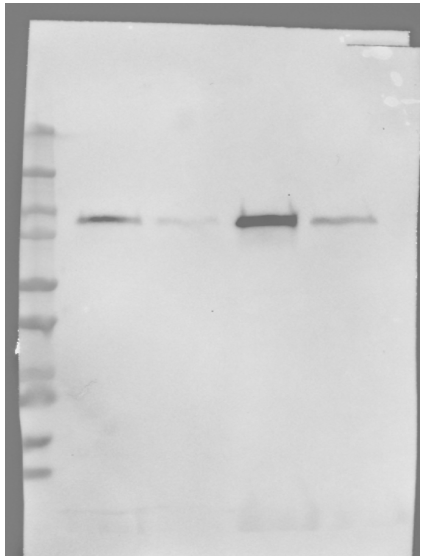

Fig. 5I: WAVE3

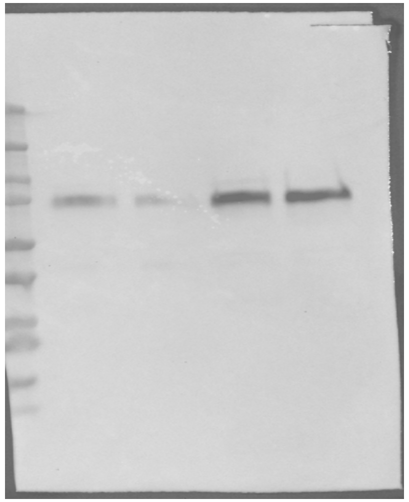

Fig. 5I: α-Tubulin

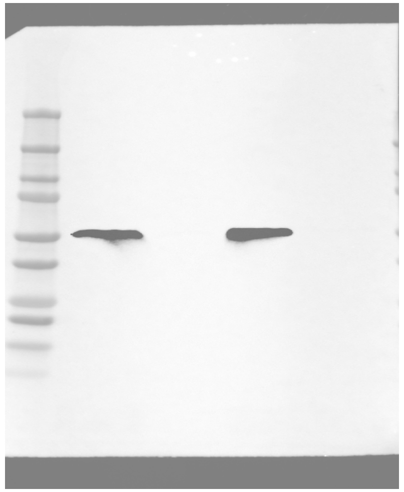

Fig. 5I: Lamin B1

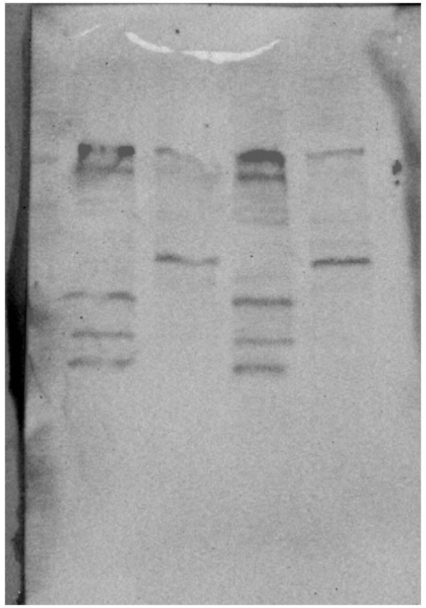

Figure 5, continued

Fig. 5J: β-Catenin

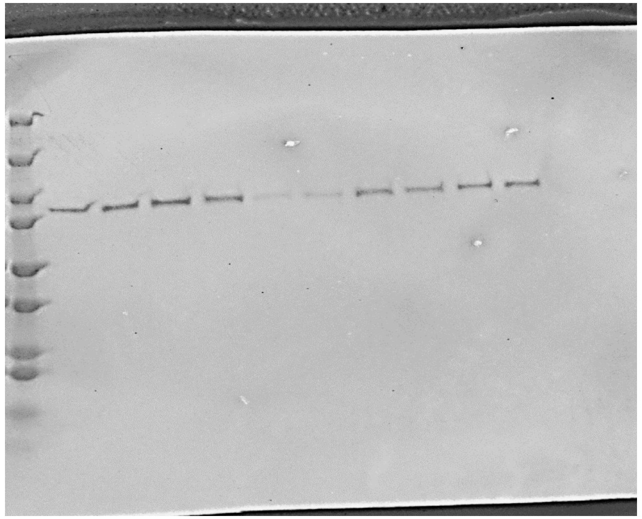

Fig. 5K: β-Catenin

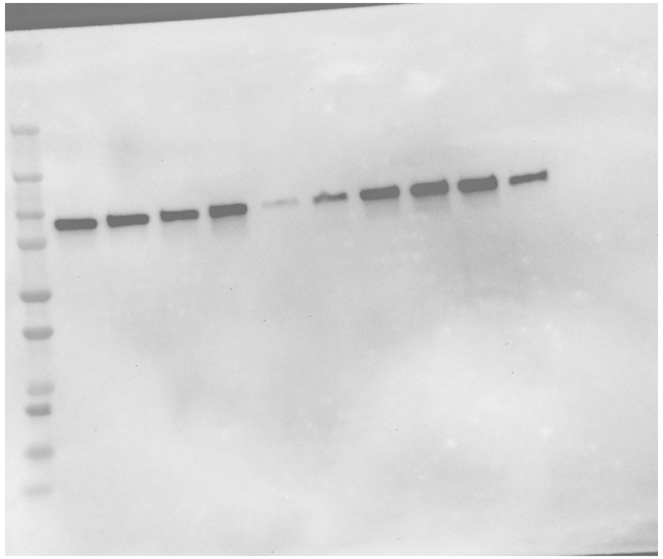

Fig. 5J: Lamin B1

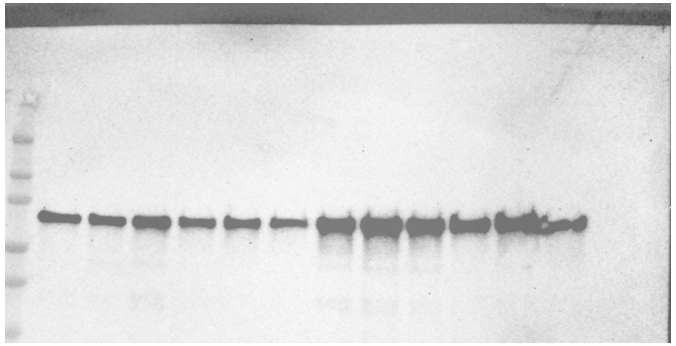

Fig. 5K: α-Tubulin

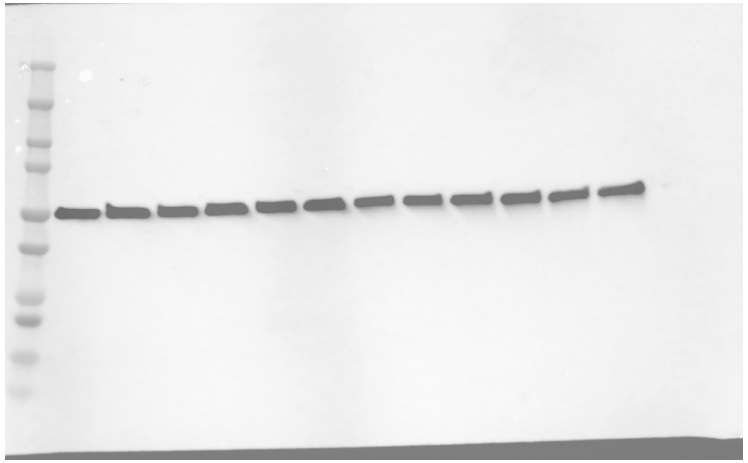

# Figure 6

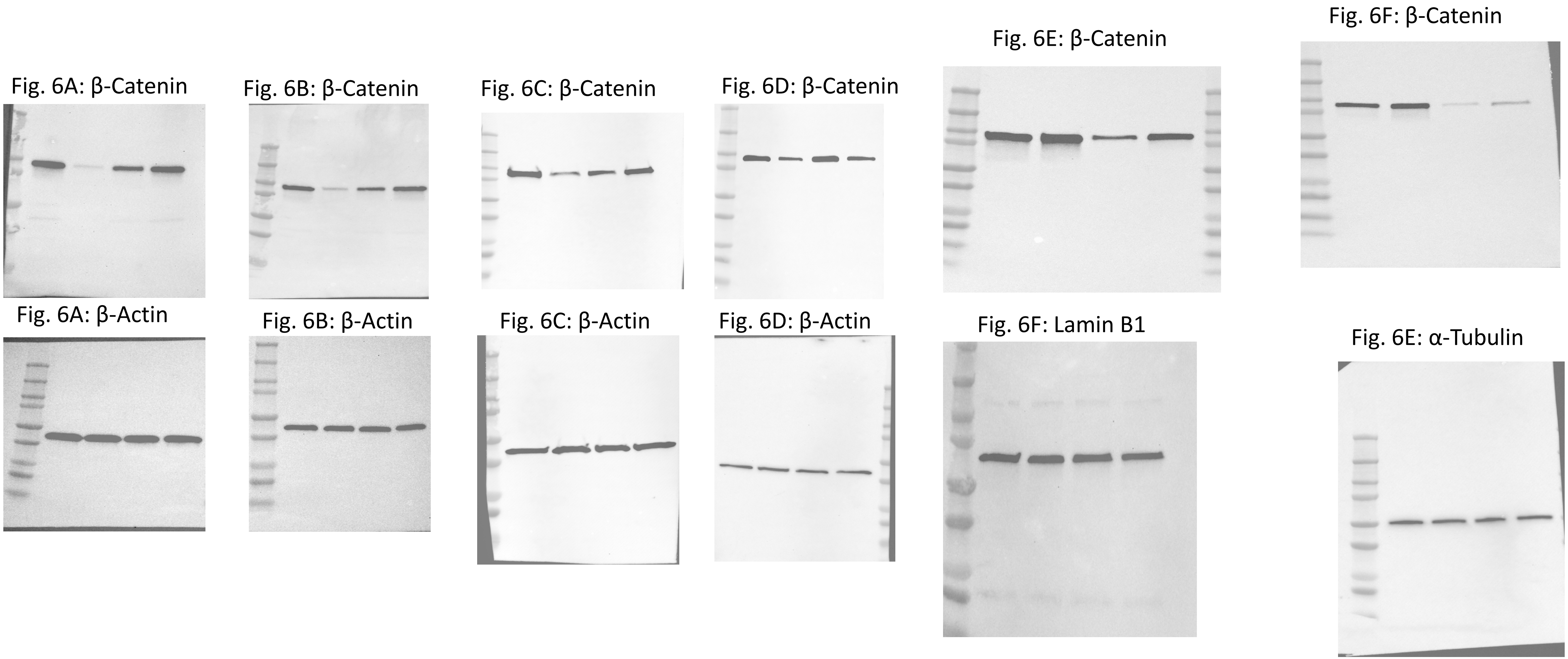
Fig. 6A: β-Catenin
Fig. 6B: β-Catenin
Fig. 6C: β-Catenin
Fig. 6D: β-Catenin
Fig. 6E: β-Catenin
Fig. 6F: β-Catenin
Fig. 6A: β-Actin
Fig. 6B: β-Actin
Fig. 6C: β-Actin
Fig. 6D: β-Actin
Fig. 6F: Lamin B1
Fig. 6E: α-Tubulin

# Figure 7

Fig. 7A: WAVE3

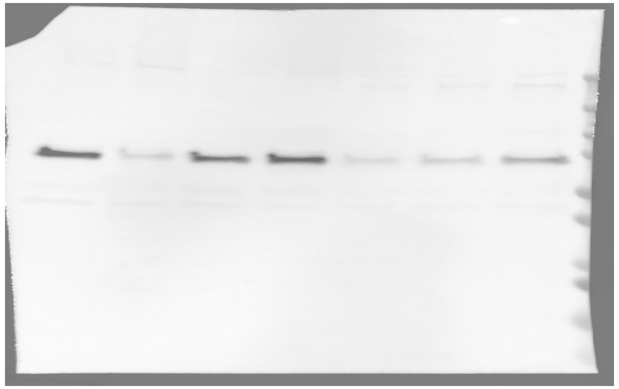

Fig. 7A: β-Catenin

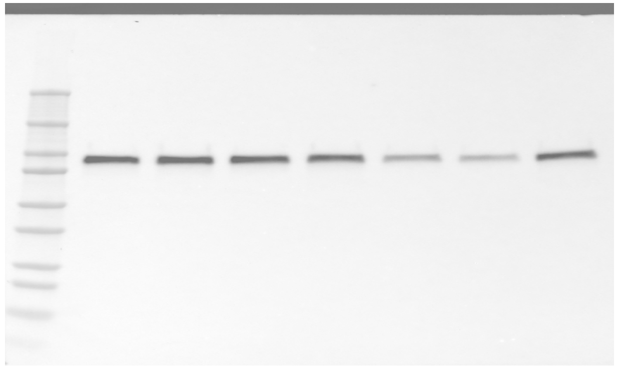

Fig. 7A: β-Actin

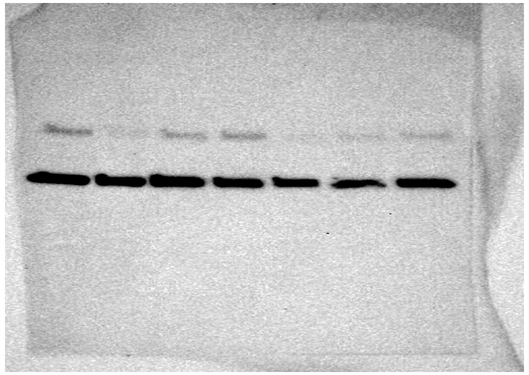

Figure S1

Fig. S1B: WAVE3

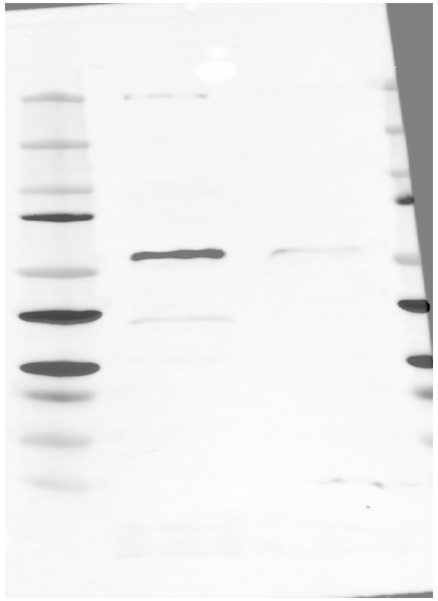

Fig. S1B: β-Actin

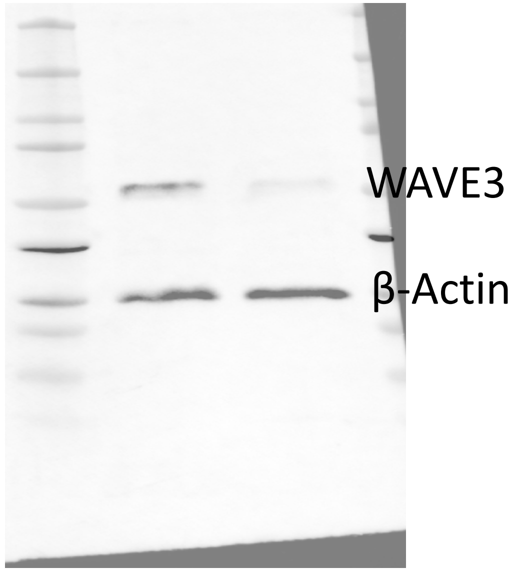


Fig. S1C: WAVE3

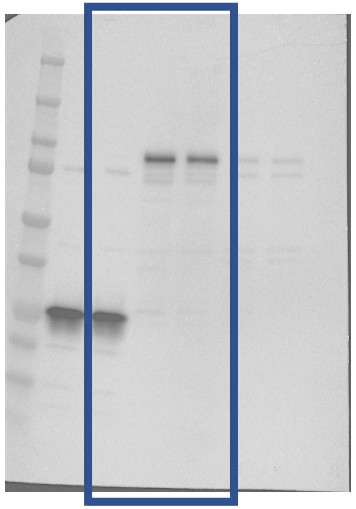

Fig. S1C: β-Actin

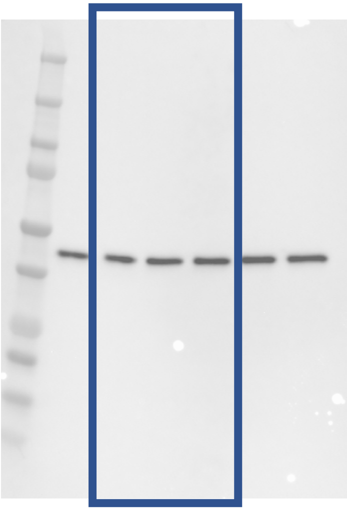

Supplement: Supplementary file 2 — Additional file 2: Contains uncropped Western Blots. [file 13058_2023_1634_MOESM2_ESM.pdf]
